# A clock-dependent brake for rhythmic arousal in the dorsomedial hypothalamus

**DOI:** 10.1038/s41467-023-41877-4

**Published:** 2023-10-11

**Authors:** Qiang Liu, Benjamin J. Bell, Dong Won Kim, Sang Soo Lee, Mehmet F. Keles, Qili Liu, Ian D. Blum, Annette A. Wang, Elijah J. Blank, Jiali Xiong, Joseph L. Bedont, Anna J. Chang, Habon Issa, Jeremiah Y. Cohen, Seth Blackshaw, Mark N. Wu

**Affiliations:** 1 Department of Neurology, Johns Hopkins University, Baltimore, MD 21205 USA; 2 McKusick-Nathans Department of Genetic Medicine, Johns Hopkins University, Baltimore, MD 21287 USA; 3 Solomon H. Snyder Department of Neuroscience, Johns Hopkins University, Baltimore, MD 21205 USA; 4 Danish Research Institute of Translational Neuroscience, Nordic EMBL Partnership for Molecular Medicine, Aarhus University, Aarhus, Denmark; 5 Department of Biomedicine, Aarhus University, Aarhus, Denmark; 6 Department of Anatomy, University of California, San Francisco, San Francisco, CA 94158 USA; 7 Biochemistry, Cellular and Molecular Biology Program, Johns Hopkins University, Baltimore, MD 21205 USA; 8 Allen Institute for Neural Dynamics, Seattle, WA 98109 USA

**Keywords:** Circadian rhythms and sleep, Molecular neuroscience

## Abstract

Circadian clocks generate rhythms of arousal, but the underlying molecular and cellular mechanisms remain unclear. In *Drosophila*, the clock output molecule WIDE AWAKE (WAKE) labels rhythmic neural networks and cyclically regulates sleep and arousal. Here, we show, in a male mouse model, that mWAKE/ANKFN1 labels a subpopulation of dorsomedial hypothalamus (DMH) neurons involved in rhythmic arousal and acts in the DMH to reduce arousal at night. In vivo Ca^2+^ imaging reveals elevated DMH^mWAKE^ activity during wakefulness and rapid eye movement (REM) sleep, while patch-clamp recordings show that DMH^mWAKE^ neurons fire more frequently at night. Chemogenetic manipulations demonstrate that DMH^mWAKE^ neurons are necessary and sufficient for arousal. Single-cell profiling coupled with optogenetic activation experiments suggest that GABAergic DMH^mWAKE^ neurons promote arousal. Surprisingly, our data suggest that mWAKE acts as a clock-dependent brake on arousal during the night, when mice are normally active. mWAKE levels peak at night under clock control, and loss of mWAKE leads to hyperarousal and greater DMH^mWAKE^ neuronal excitability specifically at night. These results suggest that the clock does not solely promote arousal during an animal’s active period, but instead uses opposing processes to produce appropriate levels of arousal in a time-dependent manner.

## Abstract (web summary)

How the circadian clock generates rhythms of arousal remains unclear. Here, authors show that a clock-output molecule reduces excitability of an arousal circuit during the active phase. These results suggest a multifaceted role for the clock in arousal.

## Introduction

Animals organize their behavior and physiology in ~24 h periods to enhance survival and fitness. Many of these oscillatory processes are driven by an internal biological clock, which allows organisms to anticipate, rather than react to, changes in the environment^1–3^. Arguably the most prominent rhythmic behavior is the daily cycling of sleep and arousal states. However, the molecular and cellular mechanisms generating rhythmic arousal remain poorly understood.

In mammals, the master circadian pacemaker is housed in the suprachiasmatic nucleus (SCN), which is essential for rhythms of sleep and wakefulness^4,5^. In addition, the dorsomedial hypothalamus (DMH) is thought to play an important role in the circadian regulation of arousal. For example, large lesions of the DMH markedly attenuate the cycling of wakefulness and locomotion^6^. The DMH has been proposed to serve as a relay between the SCN and downstream sleep/wake circuits, receiving inputs from the SCN and projecting to the locus coeruleus (LC), lateral hypothalamus (LH), and the ventrolateral preoptic nucleus (VLPO)^6–8^. While a few studies have indicated a role for DMH neuronal clusters in modulating sleep/wake^9,10^, only a single subpopulation (orexinergic DMH neurons)^11^, has been previously implicated in regulating rhythmic arousal.

At a molecular level, the circadian core oscillator controls the cycling of thousands of genes^12^. However, only a handful of genes have been suggested to regulate arousal in a clock-dependent manner, and each of these is thought to amplify behavioral rhythms by inhibiting arousal during the animal’s inactive phase^13–18^. We previously identified Wide Awake (WAKE) in *Drosophila* and demonstrated its role as a clock output molecule mediating rhythmic changes in sleep/wake states. WAKE appears to function as a dynamic intracellular scaffold, organizing signaling complexes to modulate neuronal activity in a time-dependent manner^19,20^. In line with its function in promoting cyclical behavioral states, WAKE and its mammalian homolog (mWAKE) are enriched in circadian clock neurons in both flies and mice^19–21^. Intriguingly, mWAKE is also expressed in specific brain regions outside of the SCN^21^, many of which appear to exhibit cyclical excitability^22^, suggesting that mWAKE may label rhythmic neural networks more broadly.

Here, we use mWAKE as a genetic entrée to investigate the molecular and circuit basis of rhythmic arousal. We show that mWAKE labels a subset of DMH neurons that exhibits increased activity during wakefulness and rapid eye movement (REM) sleep. Chemogenetic activation and inhibition of DMH^mWAKE^ neurons increases and decreases wakefulness, respectively. Single-cell profiling followed by intersectional optogenetic manipulations suggest that GABAergic DMH^mWAKE^ neurons promote arousal. At the molecular level, mWAKE levels rise at night in a clock-dependent manner to suppress intrinsic excitability at that time, and constitutive knockout of mWAKE produces hyperarousal at night. DMH^mWAKE^ neurons exhibit increased firing rates at night, and loss of mWAKE further elevates DMH^mWAKE^ spiking specifically at night. Finally, we find that mWAKE is required in the DMH to suppress arousal at night. Together, these findings suggest that mWAKE acts in the DMH as a rhythmic brake on neuronal excitability and arousal during the animal’s active phase. These observations argue that the circadian clock does not simply promote arousal during the night in mice, but instead generates nuanced and multi-faceted signaling to shape the appropriate level of arousal for a given circadian time.

## Results

### DMH^mWAKE^ neuronal activity is associated with wakefulness and REM sleep

WAKE is conserved from flies to humans (Supplementary Fig. 1a), and our published and ongoing work in *Drosophila* and mice suggest that WAKE labels rhythmic neural networks^19,20^. The DMH has been implicated in the circadian control of arousal, and mWAKE (also named ANKFN1/Nmf9)^23^ is expressed in this region in mice^21^. Thus, we hypothesized that DMH^mWAKE^ neurons constitute a key rhythmic arousal-promoting cell population. To address the percentage of DMH neurons that are mWAKE^+^, we performed immunostaining of DMH tissue, labeling neurons with anti-NeuN antibodies and mWAKE using anti-V5 antibodies in a validated mWAKE^V5^ transgenic mouse line^21^. These experiments suggest that DMH^mWAKE^ neurons comprise 11.6 ± 0.2% (*n* = 3 brains, 11 total sections) of the total population of DMH neurons (Supplementary Fig. 1b).

**Fig. 1:**
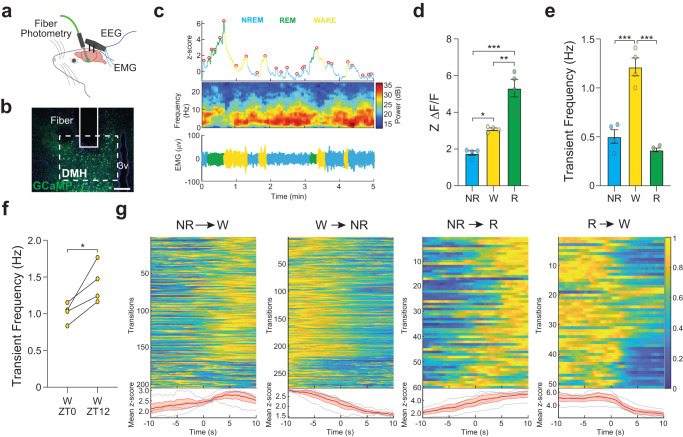
In vivo Ca^2+^ imaging of DMH^mWAKE^ neurons reveals greater activity during wakefulness and REM sleep. **a** Schematic showing the in vivo fiber photometry configuration, with simultaneous EEG/EMG recording. **b** Representative confocal image of the DMH following injection of AAV-Flex-GCaMP6s virus. The DMH and location of the fiber are indicated by dashed and solid lines, respectively. Scale bar denotes 200 μm. **c** Representative traces of a 5 min fiber photometry recording combined with EEG and EMG recordings. Top row shows the GCaMP6s signal, middle row indicates the EEG power spectrogram, and bottom trace shows the EMG signal across spontaneous sleep and wake stages. Colors correspond to distinct sleep-wake stages as shown. Circles indicate the transient peaks detected by the peak-finding algorithm (see Methods). **d** Z-score of ΔF/F signal across non-REM (NR), wakefulness (W), and REM (R) stages. In this plot, data from ZT0-3 and ZT12-15 were pooled (*n* = 4 animals); one-way ANOVA with post-hoc Tukey, ***P < 0.0001 (NR-R), **P = 0.001 (W-R), *P = 0.027 (NR-W). **e** Mean transient frequency across sleep-wake stages (*n* = 4 animals); one-way ANOVA with post-hoc Tukey, ***P < 0.0001 (NR-W), ***P < 0.0001 (W-R). **f** Mean transient frequency for wake state compared between ZT0-3 and ZT12-15 (*n* = 4 animals); paired t-test with Holm-Bonferroni correction, *P = 0.0379, two-tailed. **g** Top panels show heatmap representation of z-scored fluorescence for transitions for NREM→WAKE (*n* = 205), WAKE→NREM (*n* = 277), NREM→REM (*n* = 60), and REM→WAKE (*n* = 51). Bottom panels show averaged transition traces, shading indicates SEM. The data in this figure were derived from 4 animals, with 1–2 recording days per animal (both ZT0-3 and ZT12-15 for each day). Error bars, SEM.

Next, we investigated how the population activity of DMH^mWAKE^ neurons correlates with specific sleep/wake states in vivo. To do this, we conducted simultaneous fiber photometry/electroencephalography (EEG) recordings during ZT0-3 (Zeitgeber time 0-3) and ZT12-15. To genetically target mWAKE^*+*^ neurons, we generated transgenic mice where exon 5 was replaced with a tdTomato-P2A-Cre cassette (*mWake*^*Cre*^) (Supplementary Fig. 1c). We confirmed fidelity of the *mWake*^*Cre*^ expression pattern, by comparing its fluorescence to whole-brain RNAscope ISH labeling of *mWake*, as well as anti-V5 signal from *mWake*^*V5*^ mice^21^. AAV viral vector expressing Cre-dependent GCaMP6s was injected into the DMH of *mWake*^*(Cre/+)*^ mice, which were then implanted with a fiber photometry probe and EEG and electromyography (EMG) electrodes (Fig. 1a, b; Supplementary Table 1; Supplementary Fig. 1d). GCaMP signal across ZT0-3 and ZT12-15 timepoints was first pooled and analyzed across different vigilance states as determined by EEG/EMG. As shown in Fig. 1c, d, DMH^mWAKE^ activity was substantially increased during wakefulness and REM sleep, compared to non-rapid eye movement (NREM) sleep. For both states, there was no difference in DMH^mWAKE^ GCaMP signal during the day compared to the night (Supplementary Fig. 1e, f).

We next examined whether DMH^mWAKE^ Ca^2+^ transient peaks detected by fiber photometry also varied according to sleep/wake state and whether they differed between day vs night. The frequency of pooled transients was greater during wakefulness, compared to NREM and REM sleep (Fig. 1e), and the increased transients during wakefulness was more pronounced at night (Fig. 1f). In contrast, transient prominence was higher during REM sleep (Supplementary Fig. 1g). Finally, we assessed signal intensity changes across transitions between consolidated sleep/wake states (Fig. 1g). DMH^mWAKE^ GCaMP signal increased prior to the state change from NREM to wakefulness and from NREM to REM sleep and decreased prior to the state change from wakefulness to NREM sleep. In contrast, DMH^mWAKE^ GCaMP signal decreased following transitions from REM sleep to wakefulness, suggesting that these cells may be less important for driving those state changes. Collectively, these data demonstrate that the overall activity of DMH^mWAKE^ neurons is increased during wakefulness and REM sleep and that Ca^2+^ transients are elevated during wakefulness, particularly at night. These findings support the notion that DMH^mWAKE^ neurons are involved in the production of rhythmic arousal.

### DMH^mWAKE^ neurons promote arousal

**Fig. 2:**
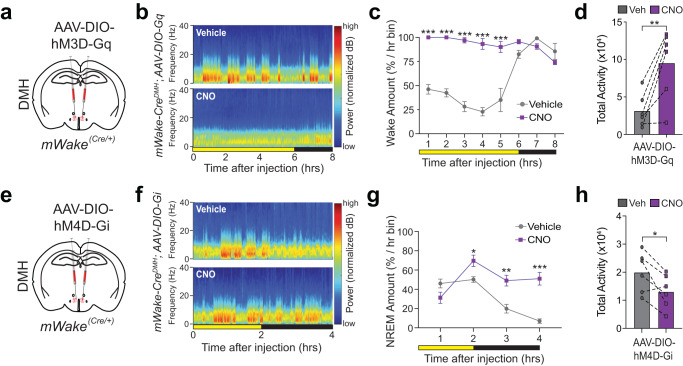
Chemogenetic manipulation of DMH^mWAKE^ neurons demonstrate a functional role in arousal. **a** Schematic showing bilateral injections of AAV-DIO-hM3D-Gq into the DMH of *mWake*^*(Cre/+)*^ mice. **b** Representative short-time Fourier transform spectrograms of 8 hrs of recorded EEG activity, starting after IP injection at ZT6 of vehicle alone (above) or 1 mg/kg CNO (below), from *mWake*^*(Cre/+)*^ mice injected with AAV-DIO-hM3D-Gq bilaterally into the DMH. Power density is represented by the color-scheme and deconvoluted by frequency on the *y*-axis and over time on the *x*-axis. **c** Amount of wakefulness derived from EEG plotted as % time in 1 h bins for the mice in (**a**), following IP injection of vehicle (gray) or 1 mg/kg CNO (magenta) (*n* = 4) at ZT6; two-way ANOVA with post-hoc Holm-Sidak, ***P < 0.0001 (1 hrs), ***P < 0.0001 (2 h), ***P < 0.0001 (3 h), ***P < 0.0001 (4 h), ***P < 0.0001 (5 h). *n* = 3 replicates. **d** Total locomotor activity (total number of beams broken along *X* and *Y*-axis) of the mice in Fig. 2a in the 4 h following IP injection of vehicle vs CNO (1 mg/kg) at CT 8 (*n* = 6); paired t-test, **P = 0.0093, two-tailed. *n* = 3 replicates. **e** Schematic showing bilateral injections of AAV-DIO-hM4D-Gi into the DMH of *mWake*^*(Cre/+)*^ mice. **f** Representative short-time Fourier transform spectrograms of 4 hrs of recorded EEG activity, starting after IP injection of vehicle alone (above) or 3 mg/kg CNO (below) at ZT10, from the mice shown in (**e**). **g** NREM amount for the mice shown in (**e**), plotted as % time in 1 h bins following IP injection of vehicle (gray) or 3 mg/kg CNO (magenta) (*n* = 6) at ZT10; two-way ANOVA with post-hoc Holm-Sidak, *P = 0.0467 (2 h), **P = 0.0026 (3 hrs), ***P < 0.0001 (4 h). *n* = 3 replicates. **h** Total locomotor activity for the mice shown in (**e**) in the 4 h following IP injection of vehicle (gray) vs CNO (3 mg/kg, magenta) (*n* = 6) at ZT10; paired *t*-test, *P = 0.0280, two-tailed. Error bars, SEM. *n* = 3 replicates.

To functionally manipulate DMH^mWAKE^ neurons, we used a chemogenetic approach by selectively expressing Designer Receptors Exclusively Activated by Designer Drugs (DREADDs)^24^ in these neurons. First, we chemogenetically activated DMH^mWAKE^ neurons by injecting an AAV vector carrying Cre-dependent DREADD-hM3Dq (AAV-DIO-hM3D-Gq)^25^ into the DMH of *mWake*^*(Cre/+)*^ mice (Fig. 2a; Supplementary Fig. 2a, b; Supplementary Table 1). CNO-mediated activation of DMH^mWAKE^ neurons at ZT6 markedly increased wakefulness, with concomitant reductions in NREM and REM sleep (Fig. 2b, c; Supplementary Fig. 2c, d). Moreover, chemogenetic activation of DMH^mWAKE^ neurons substantially increased locomotor activity, compared to vehicle-treated animals (Fig. 2d). In contrast, equivalent CNO administered to sham-injected *mWake*^*(Cre/+)*^ mice did not affect vigilance state or locomotor activity (Supplementary Fig. 2e, f).

We next examined the effects of silencing DMH^mWAKE^ neurons on sleep/wake states. We chemogenetically inhibited DMH^mWAKE^ neurons, by injecting an AAV vector encoding Cre-dependent DREADD-hM4Di (AAV-DIO-hM4D-Gi) into *mWake*^*(Cre/+)*^ mice (Fig. 2e; Supplementary Fig. 2g, h; Supplementary Table 1). Injection of CNO at ZT10 significantly increased NREM sleep, with accompanying reductions in wakefulness and, to a lesser degree, REM sleep (Fig. 2f, g; Supplementary Fig. 2i, j). Locomotor activity was also reduced with chemogenetic inhibition of DMH^mWAKE^ neurons (Fig. 2h). Together, these data suggest that DMH^mWAKE^ neurons are both necessary and sufficient for promoting arousal and wakefulness.

### GABAergic DMH^mWAKE^ neurons are arousal-promoting

**Fig. 3:**
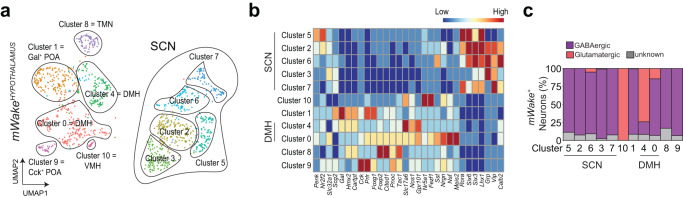
Single-cell profiling of *mWake* hypothalamic neurons. **a** UMAP (Uniform Manifold Approximation and Projection) plot showing distribution of *mWake*^+^ neurons across hypothalamic nuclei, as determined by single-cell expression profiling. 11 clusters are defined, for SCN, DMH, POA (pre-optic area), TMN (tuberomammillary nucleus), and VMH (ventromedial hypothalamus) regions. “Gal^+^” and “Cck^+^” refer to Galanin^+^ and Cholecystokinin^+^. **b** Heatmap showing key marker genes that were used to identify spatial location of each *mWake*^+^ neuronal cluster. **c** Bar graph showing proportions of GABAergic and glutamatergic *mWake*^+^ neurons for each scRNA-Seq neuronal cluster. *n* = 2 replicates.

To investigate the molecular identity of DMH^mWAKE^ cells, we conducted single-cell RNA sequencing (scRNA-Seq) of FACS-sorted tdTomato^+^ cells from the hypothalami of *mWake*^*(Cre/+)*^ mice (Supplementary Fig. 3a, b)^21^. The identity of different neuronal *mWake*^+^ clusters and the spatial location of these clusters were determined by comparison with a hypothalamic scRNA-Seq database and using specific gene markers^26^. This analysis revealed 11 *mWake*^+^ clusters in the hypothalamus, with 5 SCN-specific clusters and 2 DMH clusters (Fig. 3a, b). Collectively, hypothalamic *mWake*^+^ neurons constituted a heterogeneous group, but were generally GABAergic or glutamatergic. Among the 2 *mWake*^+^ DMH clusters, Cluster 0 was largely GABAergic, while Cluster 4 was primarily glutamatergic (Fig. 3c). In contrast, arousal-related monoaminergic and orexinergic markers were not appreciably expressed in *mWake*^+^ hypothalamic neurons, including DMH^mWAKE^ neurons.

While historically there has been substantial focus on the role of neuromodulatory circuits in regulating arousal^27,28^, accumulating evidence has highlighted the importance of both glutamatergic and GABAergic subcortical neuronal groups in this process^28–31^. Thus, we next used the INTRSECT approach^32^ to try to isolate GABAergic or glutamatergic DMH^mWAKE^ subpopulations and investigate their role in regulating arousal. We first confirmed that we could selectively manipulate GABAergic mWAKE^DMH^ neurons, by stereotactically injecting AAV-Con/Fon-EYFP virus into the DMH of *mWake*^*(Cre/+)*^*; Vgat*^*(Flp/+)*^ mice and performing immunostaining and RNAscope in situ hybridization (Supplementary Fig. 4a-c; Supplementary Table 1).

However, using a related approach for glutamatergic DMH^mWAKE^ neurons (AAV-Con/Fon-ChR2-EFYP virus with *mWake*^*(Cre/+)*^*; Vglut2*^*(Flp/+)*^ mice) resulted in little to no expression in the DMH (Supplementary Fig. 4d; Supplementary Table 1). Thus, we next tried injecting AAV-Con/Foff-EFYP virus into the DMH of *mWake*^*(Cre/+)*^*; Vgat*^*(Flp/+)*^ mice, but found in this case that viral expression was observed in substantial numbers of both glutamatergic and GABAergic DMH^mWAKE^ neurons (Supplementary Fig. 4f, g; Supplementary Table 1). We further tried to address this technical limitation by employing the recently developed rTARGIT system, where a tetracycline transactivator (tTA) is used to boost expression in a dual recombinase system (AAV-hSYN1-fDIO-rTTA and AAV-TRE-DIO-ChR2-EYFP with *mWake*^*(Cre/+)*^*; Vglut2*^*(Flp/+)*^ mice)^33^. However, even this approach did not yield significant expression in glutamatergic DMH^mWAKE^ neurons (Supplementary Fig. 4e; Supplementary Table 1). Thus, we were able to selectively manipulate GABAergic, but not glutamatergic, DMH^mWAKE^ neurons.

**Fig. 4:**
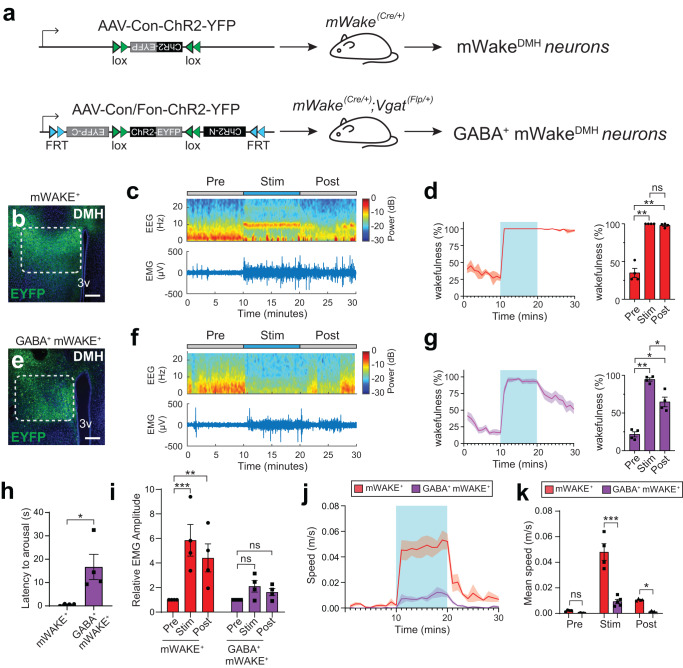
Optogenetic manipulation suggests an arousal-promoting role for GABAergic DMH^mWAKE^ neurons. **a** Schematic illustrating Cre-dependent expression of ChR2 in DMH^mWAKE^ neurons (above) and INTRSECT strategy^32^ for intersectional expression in GABAergic DMH^mWAKE^ neurons (below). **b** and **e** Representative image of native YFP fluorescence expression in the DMH (dashed outline) following injection of the viruses depicted in (**a**). Scale bar denotes 200 μm. 3 v, 3rd ventricle. **c** and **f** Representative short-time Fourier transform spectrograms of EEG activity (above) and plots of EMG amplitude (below) across 10 min before (“Pre”), 10 min during (“Stim”), and 10 min after (“Post”) 10 Hz optogenetic stimulation of DMH^mWAKE^ (**c**) or GABAergic DMH^mWAKE^ neurons (**f**) using the mice described in (**a**). Power density is represented by the color-scheme and deconvoluted by frequency on the *y*-axis, over time on the *x*-axis. **d** and **g** (left) Wakefulness plotted as % time in 1 min bins for *mWake*^*(Cre/+)*^ (**d**) and *mWake*^*(Cre/+)*^*; Vgat*^*(Flp/+)*^ mice (**g**) shown in (**a**). Optogenetic stimulation indicated by light blue box. (right) % wakefulness for the 10 min before, during, and after optogenetic stimulation of DMH^mWAKE^ neurons (red, *n* = 4 animals), one-way ANOVA with post-hoc Tukey, **P = 0.0034 (Pre-Stim), **P = 0.0061 (Pre-Post), *P* = 0.3793 (Stim-Post) (**d**) or GABAergic DMH^mWAKE^ neurons (magenta, *n* = 4 animals), one-way ANOVA with post-hoc Tukey, **P = 0.0011 (Pre-Stim), *P = 0.0216 (Pre-Post), *P = 0.0381 (Stim-Post) (**g**) neurons. **h** Latency to arousal from NREM sleep following optogenetic stimulation of DMH^mWAKE^ neurons (red, n = 4 animals) or GABAergic DMH^mWAKE^ neurons (magenta, *n* = 4 animals); unpaired *t*-test, *P = 0.0244, two-tailed. **i** Relative EMG amplitude for the 10 min before, during, and after optogenetic stimulation for DMH^mWAKE^ neurons (red, *n* = 4 animals), two-way ANOVA with post-hoc Tukey, ***P < 0.0001 (Pre-Stim), **P = 0.0018 (Pre-Post) or GABAergic DMH^mWAKE^ neurons (magenta, *n* = 4 animals), two-way ANOVA with post-hoc Tukey, *P* = 0.3393 (Pre-Stim), *P* = 0.6902 (Pre-Post). **j** Average speed (m/s) plotted per 1 min bins for activation of DMH^mWAKE^ neurons (*n* = 4 animals) or GABAergic DMH^mWAKE^ neurons (*n* = 5 animals). Optogenetic stimulation indicated by the light blue box. **k** Mean speed (m/s) for the 10 min before, during, or after optogenetic stimulation of DMH^mWAKE^ neurons (red, *n* = 4 animals) or GABAergic DMH^mWAKE^ neurons (magenta, *n* = 5 animals) in (**j**); two-way ANOVA with post-hoc Sidak’s, *P* = 0.9313 (Pre), ***P < 0.0001 (Stim), *P = 0.0447 (Post). Shading denotes SEM. Error bars, SEM.

First, to confirm our previous findings derived from chemogenetic manipulations, we performed optogenetic activation of DMH^mWAKE^ neurons (10 Hz for 10 min) from ZT2-7, while simultaneously recording EEG/EMG signals and conducting video analysis of locomotion and other behaviors (Fig. 4a, b; Supplementary Fig. 5a; Supplementary Table 1). Optogenetic activation of DMH^mWAKE^ neurons rapidly and potently induced wakefulness that persisted after the cessation of the stimulation (Fig. 4c, d, h). DMH^mWAKE^ neuron activation was also accompanied by a significant increase in EMG amplitude (Fig. 4i) and rapidly triggered intense locomotion (Fig. 4j, k; Supplementary Fig. 5c, d; Supplementary Movie 1). Similar, but milder, effects were seen on wakefulness and locomotion with optogenetic activation of GABAergic DMH^mWAKE^ neurons (Fig. 4a, e-h, j, k; Supplementary Fig. 5b-d; Supplementary Movie 2), whereas EMG amplitude was not significantly affected (Fig. 4i). Interestingly, feeding was increased during optogenetic stimulation of GABAergic DMH^mWAKE^ neurons, which was not observed during activation of all DMH^mWAKE^ neurons (Supplementary Fig. 5e).

We next analyzed the projection patterns for DMH^mWAKE^ vs GABAergic DMH^mWAKE^ neurons in these mice (Supplementary Fig. 6). Broadly speaking, the GABAergic subset appeared to target similar regions as DMH^mWAKE^ neurons as a whole, with many of these regions implicated in the regulation of arousal and sleep (i.e., preoptic area/POA, lateral hypothalamus/LH, dorsomedial hypothalamus/DMH, paraventricular thalamic nucleus/PV, lateral habenula/LHb, ventral tegmental area/VTA, posterior hypothalamus/PH, periaqueductal gray/PAG, and locus coeruleus/LC)^30,34–36^ (Fig. 4b, e; Supplementary Fig. 6a). However, there were a few notable differences; GABAergic DMH^mWAKE^ neurons exhibited absent or substantially weaker projections to the zona incerta (ZI), medial mammillary (MM) region, and the raphe pallidus nucleus (RPA) (Supplementary Fig. 6b-g). Taken together, these data suggest that GABAergic DMH^mWAKE^ neurons promote arousal.

### mWAKE suppresses arousal during the active period in mice

**Fig. 5:**
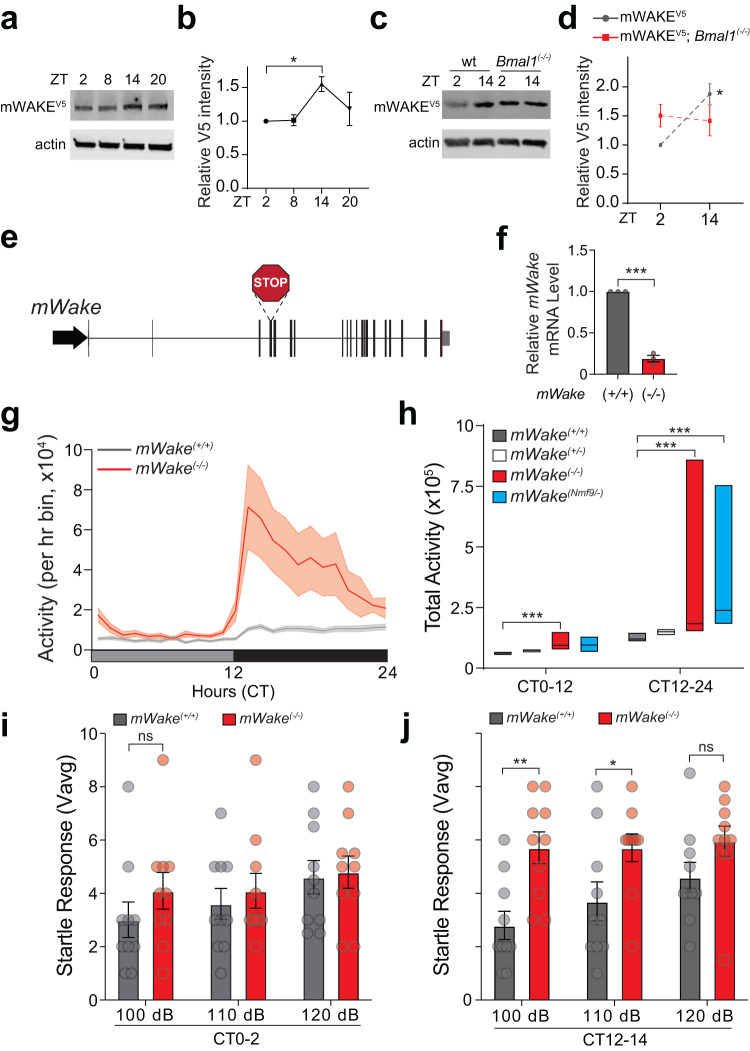
mWAKE cycles under clock control and acts to suppress arousal at night. **a**, **b** Representative immunoblot (**a**) and relative levels of mWAKE-V5 (**b**) from Western blot analyses using anti-V5 antibodies for *mWake*^*(V5/V5)*^ hypothalamic tissue at ZT2, ZT8, ZT14, and ZT20 (*n* = 4 for all time points); one-way ANOVA with post-hoc Dunnett, *P = 0.043 (ZT2-ZT14). Actin was used as a loading control. **c** and **d** Representative immunoblot (**c**) and relative levels of mWAKE-V5 (**d**) from Western blot analyses using anti-V5 antibodies at ZT2 or ZT14 for *mWake*^*(V5/V5)*^ (“wt”) (*n* = 4 for both time points) or *mWake*^*(V5/V5)*^*; Bmal1*^*(-/-)*^ (*n* = 4 for both time points) hypothalamic tissue; two-way ANOVA with post-hoc Sidak, *P = 0.023 (*mWake*^*(V5/V5)*^, ZT2-ZT14). **e** Schematic showing genomic structure of the *mWake* locus and CRISPR/Cas9-mediated insertion of 8 bp containing an in-frame stop codon in exon 4 in the *mWake*^*(-)*^ mutation. **f** Relative mRNA level for *mWake*, determined by qPCR, in *mWake*^*(-/-)*^ vs WT littermate control hypothalami (normalized to 1.0) (*n* = 3 replicates); unpaired *t*-test, ***P < 0.0001, two-tailed. **g** Profile of locomotor activity (defined by beam-breaks) over 24 h for *mWake*^*(+/+)*^ (gray) and *mWake*^*(-/-)*^ (red) mice under DD conditions. Shading denotes SEM. **h** Total locomotor activity from CT0-12 and CT12-24 from *mWake*^*(+/+)*^ (*n* = 19, gray), *mWake*^*(+/-)*^ (*n* = 22, white), *mWake*^*(-/-)*^ (*n* = 19, red), and *mWake*^*(Nmf9/-)*^ (*n* = 9, cyan) mice under DD conditions; Kruskal-Wallis test with post-hoc Dunn’s, ***P = 0.0001 (CT0-12: *mWake*^*(+/+)*^*-mWake*^*(-/-)*^*)*, ***P = 0.0007 (CT12-24: *mWake*^*(+/+)*^*-mWake*^*(-/-)*^*)*, ***P = 0.0007 (CT12-24: *mWake*^*(+/+)*^*-mWake*^*(Nmf9/-)*^*)*. Simplified boxplots show 25th percentile, median, and 75^th^ percentile. n = 4 replicates. **i** and **j** Startle response (Vavg) measured in the first 100 ms following a 100, 110, or 120 dB tone for *mWake*^*(+/+)*^ (gray, *n* = 10) vs *mWake*^*(-/-)*^ mice (red, *n* = 10) at CT0-2 (**i**) or CT12-14 (**j**); two-way ANOVA with repeated measures and post-hoc Holm-Sidak, *P* = 0.5417 (CT0: 100 dB), **P = 0.003 (CT12: 100 dB), *P = 0.0391 (CT12: 110 dB), *P* = 0.1106 (CT12: 120 dB). The average of 5 responses is shown. Error bars, SEM. *n* = 4 replicates.

Our data suggest that DMH^mWAKE^ neurons promote arousal, but what is the function of the mWAKE molecule itself in arousal? In *Drosophila*, WAKE cycles under clock control and is required for the rhythmic modulation of sleep/wake states^19,20^. We thus asked whether mWAKE plays a similar role in mice. Using Western blot analyses from *mWake*^*(V5/V5)*^ mice, we first examined mWAKE expression in hypothalamic tissue at different times of day. mWAKE levels peak in the early night, and this cycling is dependent on intact clock function, as it is lost in a *Bmal1* mutant background (Fig. 5a–d).

To test the function of mWAKE on sleep and arousal, we generated a null allele of *mWake* (*mWake*^*(-)*^) using CRISPR/Cas9 (Fig. 5e and see Methods). As expected, *mWake* expression, as assessed by quantitative PCR (Fig. 5f) and in situ hybridization (ISH)^21^, was markedly reduced in *mWake*^*(-/-)*^ mice, likely due to nonsense-mediated decay. We next assessed sleep in *mWake*^*(-/-)*^ mice via EEG/EMG recordings. Under light:dark (LD) or constant dark (DD) conditions, there was either subtle or no differences in the amount of wakefulness, NREM, or REM sleep between *mWake*^*(-/-)*^ mutants and wild-type (WT) littermate controls (Supplementary Fig. 7a, b). However, there was a change in the distribution of wakefulness at night; mutants spent more daily time in prolonged wake bouts, with some (33%) exhibiting dramatically long (>6 h) bouts of wakefulness (Supplementary Fig. 7c, d). These data suggest that *mWake* mutants exhibit changes in the quality, but not quantity, of wakefulness at night.

Alterations in arousal level can be quantified across different parameters, including sleep/wake behavior, locomotor activity, and responsiveness to sensory stimuli^37^. Thus, we next examined baseline homecage locomotor activity. Surprisingly, *mWake*^*(-/-)*^ mutants were markedly hyperactive during the subjective night compared to littermate controls, although a mild but significant increase in locomotor activity was also noted during the subjective day (Fig. 5g, h and Supplementary Movie 3). To rule out the possibility of 2nd site mutations causing this phenotype, we examined heteroallelic *mWake*^*(Nmf9/-)*^ mutants, which also demonstrated robust locomotor hyperactivity during the night, but not during the day (Fig. 5h). Under LD conditions, *mWake*^*(-/-)*^ and *mWake*^*(Nmf9/-)*^ mice also demonstrated increased locomotor activity during the night, although a mild, but statistically significant increase in locomotion was also seen in *mWake*^*(-/-)*^ mice during the daytime (Supplementary Fig. 8a, b). Locomotor activity for heterozygous *mWake*^*(+/-)*^ mice was not different from littermate controls (Fig. 5h). The variability of the pronounced nighttime locomotor activity in *mWake*^*(-/-)*^ and *mWake*^*(Nmf9/-)*^ mutants was driven by intense stereotypic circling behavior in a subset (~35%) of these animals (Supplementary Movie 3). These “circlers” tended to also exhibit pronounced theta activity on EEG spectrograms (Supplementary Fig. 7c), which has been associated with “active” wakefulness^38^. We next characterized sensory responsiveness in *mWake*^*(-/-)*^ mutants by evaluating startle response to an acoustic stimulus during the subjective day and subjective night. *mWake*^*(-/-)*^ mutants exhibited an increased startle response to 100 dB and 110 dB stimuli during subjective night, but not during the subjective day (Fig. 5i, j). Taken together, these data suggest that the clock acts through mWAKE to paradoxically attenuate arousal during the active period of mice.

### mWAKE acts in the DMH as a rhythmic brake on arousal by reducing neuronal excitability at night

**Fig. 6:**
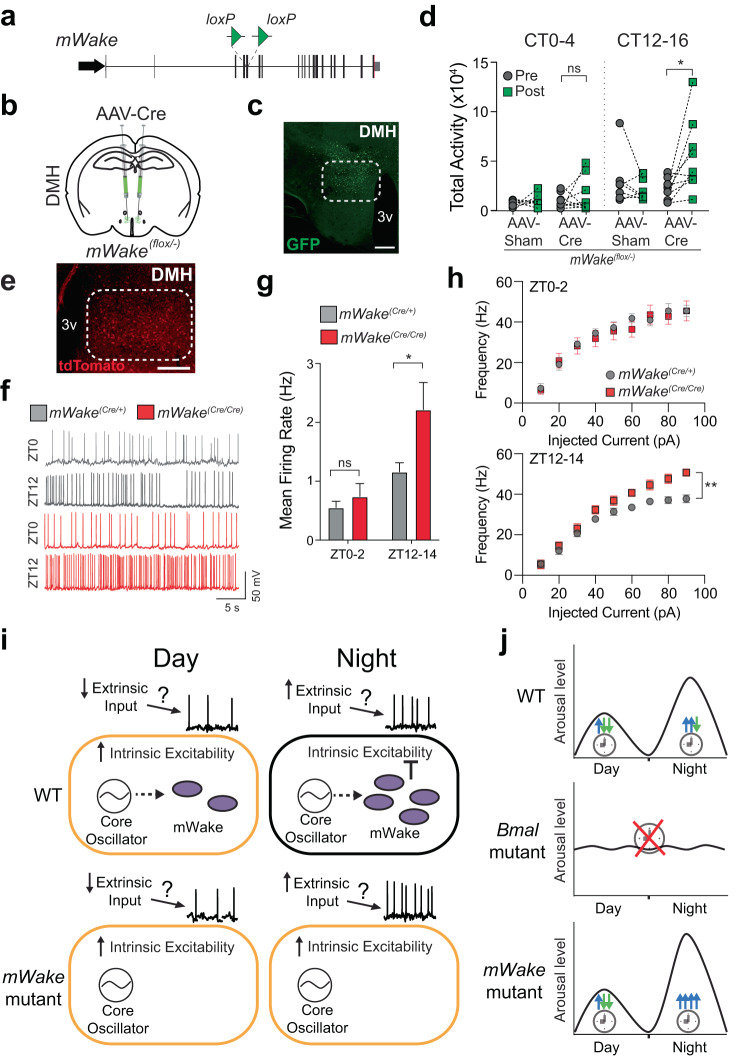
mWAKE is required in the DMH to modulate arousal and inhibits the excitability and firing of DMH neurons at night. **a** Schematic of the genomic structure of the *mWake* locus and insertion of *loxP* sites flanking exon 5 in the *mWake*^*(flox)*^ allele. **b** Schematic showing bilateral injections of AAV viral vector containing Cre-recombinase and GFP (AAV-Cre), or GFP alone (AAV-Sham) into the DMH of *mWake*^*(flox/-)*^ mice. **c** Representative image of GFP fluorescence expression in the DMH following AAV-Cre injection described in (**a**). Scale bar, 200 μm. **d** Total locomotor activity during CT0-4 vs CT12-16 under DD conditions for *mWake*^*(flox/-)*^ mice before (“pre”, gray) and after (“post”, green) DMH injection of AAV-Sham (*n* = 7) or AAV-Cre (*n* = 9); paired t-test with Holm-Bonferroni correction, *P* = 0.3995 (CT0-4: AAV-Cre), *P = 0.0448 (CT12-16: AAV-Cre), two-tailed. *n* = 4 replicates. **e** Native tdTomato fluorescence in the DMH of a *mWake*^*(Cre/+)*^ mouse (dashed lines denote DMH region). Scale bar represents 200 μm. 3 v, 3rd ventricle. **f** Representative membrane potential traces from whole-cell patch clamp recordings of DMH^mWAKE^ neurons in *mWake*^*(Cre/+)*^ (gray, top) and *mWake*^*(Cre/Cre)*^ (red, bottom) slices at ZT0-2 and ZT12-14. **g** Spontaneous mean firing rate for DMH^mWAKE^ neurons at ZT0-2 and ZT12-14 from *mWake*^*(Cre/+)*^ (*n* = 18 and *n* = 19, gray) vs *mWake*^*(Cre/Cre)*^ (*n* = 15 and *n* = 11, red) mice; unpaired *t*-test with Holm-Bonferroni correction, *P* = 0.4283 (ZT0), *P = 0.0268 (ZT12), two-tailed. **h** *f*-*I* curves for DMH^mWAKE^ neurons from *mWake*^*(Cre/+)*^ (gray) vs *mWake*^*(Cre/Cre)*^ (red) mice at ZT0-2 (*n* = 11 and 11, top) or ZT12-14 (*n* = 8 and 8, bottom); two-way ANOVA with repeated measures, **P = 0.0024 (ZT12-14) For panels (**g**) and (**h**), *n* represents individual cells from ≥4 animals for each condition. Error bars, SEM. **i** Model. mWAKE levels are higher at night, leading to reduced intrinsic excitability of DMH^mWAKE^ neurons at night. However, putative increased extrinsic inputs onto these cells induced increased spontaneous firing of DMH^mWAKE^ neurons at night. Loss of mWAKE leads to greater intrinsic excitability selectively at night, which further enhances spontaneous firing at night. **j** Model. While the clock promotes arousal at night in mice, it generates both arousal-promoting and arousal-inhibiting signals during this time. Loss of core clock activity (e.g., *Bmal* mutant) leads to loss of rhythms of arousal^50^. In contrast, loss of mWAKE selectively removes the clock-dependent arousal-inhibiting signal at night, leading to marked hyperarousal at night.

Because our data suggest that DMH^mWAKE^ neurons promote arousal, we next investigated whether mWAKE functions in these cells to regulate arousal in a time-dependent manner. We generated *mWake*^*(flox/+)*^ mice by introducing loxP sites flanking exon 5 using homologous recombination (Fig. 6a). We then performed conditional knockout of mWAKE by stereotaxic injection of AAV-Cre into the DMH of *mWake*^*(flox/-)*^ mice (Fig. 6b, c; Supplementary Fig. 9a; Supplementary Table 1). Reduction of mWAKE in the DMH significantly increased locomotor activity during subjective nighttime, but not subjective daytime, compared to baseline. In contrast, no differences in locomotor activity were observed in sham-injected controls during the subjective day or night (Fig. 6d). These data suggest that mWAKE acts in DMH neurons to inhibit arousal specifically at night.

In *Drosophila*, WAKE labels rhythmic neurons and is required for their cyclical activity^19,20^. Thus, we postulated that mWAKE regulates the excitability of DMH^mWAKE^ neurons in a time-dependent manner. To address this hypothesis using electrophysiological approaches, we needed a genetic method to visualize *mWake*^*+*^ neurons while simultaneously generating a mutant *mWake* allele. Our *mWake*^*Cre*^ line labels *mWake*^*+*^ neurons^21^ and should function as a null allele, because exon 5 is replaced by a tdTomato-P2A-Cre cassette. To test this, we assessed locomotor activity of *mWake*^*(Cre/Cre*)^ animals vs heterozygous controls in DD and found that the homozygous animals phenocopied the nighttime hyperactivity of *mWake*^*(-/-)*^ and *mWake*^*(Nmf9/-)*^ mutants, demonstrating that *mWake*^*Cre*^ is a bona fide *mWake* mutant allele (Supplementary Fig. 9b, c).

We performed whole-cell patch-clamp slice recordings and found that DMH^mWAKE^ neurons exhibited greater spiking frequency during the night vs the day, a rhythm which is aligned with the active phase of the nocturnal mouse (0.55 ± 0.10 Hz at ZT0-2 vs 1.16 ± 0.15 Hz at ZT12-14 in *mWake*^*(Cre/+)*^, *P* < 0.01). Interestingly, intrinsic excitability of these neurons was reduced during the night (Supplementary Fig. 9d). Loss of mWAKE did not significantly alter spontaneous firing of DMH^mWAKE^ neurons during the daytime. However, the nighttime increase in spiking frequency seen in controls was markedly enhanced in *mWake*^*(Cre/Cre)*^ mutants (Fig. 6e–g and Supplementary Table S2). Similarly, compared to heterozygous controls, intrinsic excitability of DMH^mWAKE^ neurons was greater in *mWake*^*(Cre/Cre)*^ mutants during the night, but not the day (Fig. 6h). Together, these findings suggest that mWAKE acts in the DMH as a brake on arousal at night, by inhibiting neural excitability specifically during that time.

## Discussion

Although it is widely accepted that the circadian clock regulates arousal, the molecular and circuit mechanisms underlying this process remain poorly understood. Here, we define a sub-population of DMH neurons that functions in rhythmic arousal and demonstrate an unexpected role for the clock output molecule mWAKE in suppressing arousal during the animal’s active phase. Chemogenetic and optogenetic experiments indicate that DMH^mWAKE^ neurons promote arousal, and patch-clamp electrophysiology data suggest that the activity of these cells is greater at night when mice are active. Moreover, we find that mWAKE normally suppresses the activity of DMH^mWAKE^ neurons specifically at night to reduce arousal during this time.

Our in vivo Ca^2+^ imaging and chemogenetic inhibition data also suggest that at least some DMH^mWAKE^ neurons regulate REM sleep. Previous work has described a group of REM-promoting Galanin^+^ GABAergic neurons within the DMH that projects to the RPA^9^. However, our scRNAseq data indicate that most Galanin^+^ cells within the DMH^mWAKE^ population are glutamatergic, although there are some GABAergic Galanin^+^ DMH^mWAKE^ neurons in “cluster 4” (Fig. 3b, c). While we were able to detect DMH^mWAKE^ projections to the RPA, these projections did not appear to originate from GABAergic DMH^mWAKE^ neurons (Fig. 3b, c; Supplementary Fig. 6d, g). Thus, these data suggest that DMH^mWAKE^ neurons label a distinct group of REM-promoting cells in the DMH, although we cannot exclude the possibility of overlap between *mWake*^*+*^ neurons with the aforementioned REM-promoting circuit^9^.

There are a number of parallels between our findings on mWAKE in mice and our previously described work on WAKE in flies^19,20^. In both flies and mice, WAKE levels rise at night under clock control to suppress intrinsic excitability and spontaneous firing at that time, suggesting that the basic function of WAKE molecules is conserved across evolution. In addition, in both flies and mice, WAKE molecules are expressed in neural circuits that promote arousal (large ventrolateral neurons and DN1p neurons in flies and DMH^mWAKE^ neurons in mice). However, there is a key difference related to WAKE’s impact on behavior between flies and mice, owing to their different temporal niches (diurnal vs nocturnal). In flies, WAKE suppresses arousal circuits to promote sleep at night, whereas in mice, mWAKE inhibits arousal circuits to attenuate arousal at night (when mice are normally awake). The latter finding is counter-intuitive and raises the question of why the clock would inhibit arousal during a time when the animal is normally active.

The most widely held view is that the clock promotes wakefulness during an animal’s active period and sleep during its inactive period^5,14^. However, this view may be too simplistic. Biological processes can have co-existing agonistic and antagonistic signaling mechanisms capable of acting together to shape responses in a time-specific or context-specific manner. For example, there are molecular and circuit mechanisms dedicated not only to learning, but also to active forgetting^39,40^, and hunger and satiety circuits are often intermingled and can signal to shared targets^41,42^. Interestingly, both hunger and satiety are thought to be under circadian control^43,44^, and satiety-promoting (MSH^+^) neurons in the arcuate nucleus exhibit SCN-dependent rhythmic activity, peaking at the late night, when rats normally exhibit a feeding peak^45^. This finding suggests that there may be distinct clock-dependent circuit mechanisms that oppose the broad daily rhythms of feeding.

Here, we propose that a similar phenomenon occurs in the clock-dependent generation of arousal in mice. At the cellular level, our data suggest that the clock upregulates mWAKE protein levels in DMH^mWAKE^ neurons at night, which reduces the intrinsic excitability of those neurons at that time. However, because DMH^mWAKE^ neurons function in arousal, they normally exhibit higher firing rates at night when mice are active, which likely depends on increased extrinsic inputs onto these cells during the night (Fig. 6i). Loss of mWAKE thus leads to greater intrinsic excitability at night and, combined with the increased extrinsic inputs at night, leads to a marked increase in DMH^mWAKE^ spiking during that time, without impacting daytime firing rates. What are the implications of these molecular and cellular rhythms for arousal behavior in mice? We suggest that in nocturnal animals such as mice, the clock on balance promotes arousal at night, but this signaling is heterogenous, with most promoting arousal and some inhibiting arousal (Fig. 6j). Complete loss of clock function leads to loss of arousal rhythms and in particular reduced arousal at night. In contrast, knockout of mWAKE (either constitutively or within DMH^mWAKE^ neurons) leads to selective loss of the clock-generated arousal-suppressing signals at night, resulting in marked hyperarousal at night, but not during the day.

Thus, our findings suggest that there are distinct clock-dependent mechanisms that can oppose the general role of the clock in arousal. Such mechanisms may be important for sculpting the temporal features of arousal or help modulate arousal level and can likely only be revealed through the analysis of individual molecules (e.g., mWAKE) or specific neural circuits. Related to this point, while the spiking activity of the SCN is broadly increased during the day, a recent study identified a subcircuit within the SCN that exhibits greater firing during the night and promotes sleep during the animal’s active phase^46^. Taken together, our findings argue against a simplistic model of the circadian clock generating monolithic molecular and cellular rhythms and reveal more nuanced mechanisms by which the clock promotes arousal in a rhythmic manner.

## Methods

### Animals

All animal procedures were approved by the Johns Hopkins Institutional Animal Care and Use Committee. All animals were group-housed and maintained with standard chow and water available *ad libitum*. Animals were raised in a common animal facility under a 14:10 h Light:Dark (LD) cycle. Animals were acclimatized to a 12:12 LD schedule for at least 7 days or 14 days prior to timed molecular experiments or behavioral assays, respectively. Animals were housed under 68-79°F and 30-70% relative humidity conditions. Unless otherwise noted, adult male mice (2–4 months) were used in all experiments. All mouse strains were backcrossed to *C57BL/6* at least seven times prior to use in behavioral experiments. Genotyping was performed either by in-house PCR and restriction digest assays, or via Taq-Man based rtPCR probes (Transnetyx). *mWake*^*V5*^ mice were previously described^21^. *mWake*^*Nmf9*^ were obtained from B. Hamilton (University of California, San Diego). *Bmal1*^*-*^ (009100), *Vglut2*^*Flp*^ (030212), and *Vgat*^*Flp*^ (029591) mice were obtained from The Jackson Laboratory.

The *mWake* null mutant allele (*mWake*^*-*^) was generated by CRISPR/Cas9 genome editing (Johns Hopkins Murine Mutagenesis Core), using a targeting guide RNA (gRNA: CGC AGA AGA ATC CTC GCA AT) to the 4th exon of *mWake* and a 136 bp oligonucleotide (AGT GCG GAC TTT CTC TGG CTC CTG TCC GCA GAA GAA TCC TCG GCG GAA TTC AAT GGG CAC GTT GTT GGT CAT GAT GGC GAT GTC CAG GGG TGT CAG CCC TTC GCT GTT CGG TGT) containing two 64 bp homology arms, surrounding an 8 bp insertion (GCGGAATT), which includes an in-frame stop codon and induces downstream frameshifts. Exon 4 is predicted to be in all *mWake* splice isoforms. The conditional *mWake* knockout allele (*mWake*^*flox*^) was generated via homologous recombination in hybrid (*129/SvEv* x *C57BL/6*) mouse embryonic stem cells (Ingenious Targeting Laboratory), using a construct containing two loxP sites and a Neomycin cassette flanking the 5^th^ exon of *mWake*. A transgenic mouse line expressing both Cre recombinase and tdTomato in *mWake*^+^ cells (*mWake*^*Cre*^) was generated via homologous recombination in hybrid (*129/SvEv* x *C57BL/6*) mouse embryonic stem cells (Ingenious Targeting Laboratory). The knock-in vector was integrated 21 bp into exon 5 of the *mWake* locus, replacing the remainder of the exon with a *tdTomato-P2A-split Cre-Neo-WPRE-BGHpA* cassette, which causes frameshifted nonsense mutations downstream and producing an *mWake* loss-of-function allele.

### Molecular biology

To quantify *mWake* transcript in the *mWake*^*(-/-)*^ mutant mice, qPCR was performed. Control and *mWake*^*(-/-)*^ mutant mice were anesthetized by the isoflurane drop method and then decapitated. Hypothalami and hindbrains were dissected at ~ZT0, and RNA was extracted using Trizol Reagent (Invitrogen). qPCR was performed using a SYBR PCR master mix (Applied Biosystems) and a 7900 Real-Time PCR system (Applied Biosystems), with the following primers, which target exon 4: *mWake*-F: 5’-CCC TAA CGG TCA GCT TTC AAG A-3’ and *mWake*-R: 5’-GAC ATG CTC CAT TCC ACT TTG TAC-3’. GAPDH was used as an internal control. Ct value was compared against regression standard curve of the same primers. 3 biological replicates were performed.

### Western blot analysis

*mWake*^*(V5/V5)*^ or *mWake*^*(V5/V5)*^; *Bmal1*^*(-/-)*^ male mice were anesthetized by the isoflurane drop method and then decapitated. Hypothalami were dissected, and protein was extracted by homogenizing the tissue in RIPA solution (Millipore Sigma, R2078) with protease inhibitor cocktail (Millipore Sigma, P8340, 1:100) and incubating for 1 h on ice. Lysates were cleared by centrifugation, and protein concentration was measured using the Pierce BCA protein assay kit (ThermoFisher Scientific, 23227). Samples were then prepared in sample buffer and, after incubating at 70 °C for 10 min, 20 μg protein was run on SDS-PAGE. Proteins were transferred to PVDF membrane for 1.5 hrs at 100 V, blocked for 1 h with Intercept Blocking Buffer (LI-COR, 927-60001), and then incubated overnight at 4 °C with rabbit V5-Tag (D3H8Q) antibody (Cell Signaling Technology, 13202, 1:2000) in blocking buffer with 0.2% Tween-20. Membranes were washed 4 times with 1xTBST (20 mM Tris, 137 mM NaCl, 0.1% Tween-20, pH 7.6) then incubated with anti-β-actin (Millipore Sigma, A5441, 1:5000) antibody for 1 h at room temperature. Following 4 additional washes with 1xTBST, membranes were incubated with anti-mouse (LI-COR, IRDye 680RD, 926-68070) and anti-rabbit (LI-COR, IRDye 800CW, 926-32211) fluorescent antibodies (1:10,000) for 1 h at room temperature. After washing 4 times with 1xTBST after the incubation, membranes were rinsed with 1xTBS and imaged using the Odyssey Fc Imaging System (LI-COR Biosciences). V5 signal was quantified using ImageJ and normalized to actin loading control.

### Immunostaining and confocal imaging

For DMH^mWAKE^ neuron immunolabeling, 10–12-week-old female *mWake*^*(V5/V5)*^ mice were deeply anesthetized and then perfused with 4% paraformaldehyde in 1x PBS (137 mM NaCl, 2.7 mM KCl, 10 mM Na_2_HPO_4_, 1.8 mM KH_2_PO_4_) at ~ZT10-12. Brains were removed, postfixed and sliced into 60 μm coronal sections using a VT1200S vibratome (Leica). Sections were washed in 1xPBS, blocked for 1 h in blocking buffer (PBS + 0.25% Triton X-100 + 5% normal goat serum), and then incubated with rabbit anti-V5 (D3H8Q) antibody (Cell Signaling Technology, 13202; 1:2000) and mouse anti-NeuN antibody (Abcam, ab104224; 1:500) in blocking buffer overnight at 4 °C. After washing with PBST (PBS + 0.3% Triton X-100), sections were incubated with goat anti-rabbit Alexa Fluor 488 (Invitrogen, A-11034; 1:1000) and goat anti-mouse Alexa Fluor 568 (Invitrogen, A-11004; 1:1000) secondary antibodies for 2 h at room temperature, washed and then mounted. For tdTomato and GFP/YFP images, native fluorescence of both fluorophores was visualized.

Images were acquired using a Zeiss LSM880, LSM800, or LSM700 confocal microscope under 10x-63x magnification, with post-hoc tiling as needed using Zen Black or Zen Blue (Zeiss). For DMH^mWAKE^ neuron quantification, images were analyzed using Imaris 9.5.

### Single cell sequencing

Seven week old, male *mWake*^*(Cre/+)*^ mice were sacrificed at ~ZT5 by cervical dislocation for single-cell RNA-Sequencing (scRNA-Seq). A modified Act-Seq^47^ method was used in conjunction with a previously described dissociation protocol^26^, with supplementation of Actinomycin D during dissociation (45 μM) and after final resuspension (3 μM), following debris removal (Debris Removal Solution (130-109-398, MACS Miltenyi Biotec)) in between. 1 mm hypothalamic sections between Bregma 0.02 mm (collecting medial and lateral preoptic area) and Bregma −2.92 mm (beginning of the supramammillary nucleus) were collected, and 2–3 mice pooled per scRNA-Seq library.

Following dissociation, tdTomato+ cells were flow-sorted using an Aria Ilu Sorter (Becton Dickinson). Between 400–1000 cells were flow-sorted per brain. Flow-sorted cells were pelleted and re-suspended in 47.6 μl resuspension media. 1 μl of flow-sorted tdTomato^+^ cells were used to quantify % of tdTomato^+^ cells with a phase-contrast microscope. Only samples containing ~99% flow-sorted tdTomato^+^ cells were processed for scRNA-Seq. The remaining 46.6 μl were used for the 10x Genomics Chromium Single Cell system (10x Genomics, CA, United States) using V3.0 chemistry per manufacturer’s instructions, generating a total of 3 libraries. Libraries were sequenced on Illumina NextSeq 500 with ~150 million reads per library (~200,000 median reads per cell). Sequenced files were processed through the CellRanger pipeline (v 3.1.0, 10x Genomics) using a custom mm10 genome (with *tdTomato-P2A-Cre-WPRE-bGH* sequence). All 3 libraries were aggregated together for downstream analysis.

Seurat V3^48^ was used to perform downstream analysis following the standard pipeline, using cells with >200 genes and 1000 UMI counts, removing *mWake-tdTomato*^+^ ependymal cells and non-*mWake*-cells (~1% of the total cluster composed of oligodendrocytes and astrocytes) using known markers genes in the initial clustering^26^. Louvain algorithm was used to generate different clusters, and spatial information (spatial location of different *mWake-tdTomato*^+^ clusters across hypothalamic nuclei) and identity of neuronal clusters were uncovered by referring to a hypothalamus scRNA-Seq database^26^. Region-specific transcription factors expressed in *mWake*^+^ neurons were used to train *mWake*-scRNA-Seq. Spatial information of different *mWake* neuronal populations was further validated by matching to the Allen Brain Atlas ISH data using cocoframer^49^, as well as matching to known *mWake* neuronal distribution across the hypothalamus. The percentage of GABAergic (*Slc32a1*^+^) and Glutamatergic (*Slc17a6*^+^) neurons within each cluster was calculated.

### Behavioral analysis

Animals were entrained to a 12:12 h LD cycle for at least 2 weeks before any locomotor or EEG-based behavioral experiments. Experimenters were blinded to animal genotype.

#### Homecage locomotor activity

Animals were separated into new individual cages with access to food and water *ad libitum* and allowed to acclimate for 4 days before data collection. Data were recorded over 2 days of 12:12 h LD and 2 days of constant darkness (DD) cycles. Locomotor activity was recorded and analyzed using the Opto M3 monitoring system with IR beams spaced 0.5 inch apart and Oxymax data-acquisition software v5.27 (Columbus Instruments). Total activity (the total number of beam breaks along the *X* and *Y*-axis) was measured in 10 s intervals. Locomotor activity profiles were generated from the 2nd day of LD or the 1st day of DD.

For *mWake* conditional knockdown experiments, analysis of homecage locomotor activity was performed as described above for *mWake*^*(flox/-)*^ mice, with data recorded over 3 days of 12:12 h LD and 3 days of DD following acclimation (“Baseline”). For *mWake* conditional knockdown, mice were randomly divided into two groups to receive stereotaxic injection of AAV-Cre virus (“Cre”) or PBS (“Sham”) into the DMH (as described below and in Supplementary Table 1). After the surgery, mice were individually housed in a new cage for 4 weeks to allow for Cre-mediated recombination to occur. Mice were then transferred individually into new cages and 3 days of 12:12 h LD and 3 days of DD homecage locomotor activity was recorded as described above.

#### Acoustic startle

Acoustic startle response was recorded using an SR-LAB Startle Response System (San Diego Instruments) apparatus, which consists of a sound-isolating cabinet containing a pressure-sensitive plate. Mice were placed into a plexiglass tube (I.D. 5 cm) and then enclosed inside the chamber on the pressure-recording plate for the duration of the trial. Mice were acclimated to the test environment, including 50 dB of background white noise, for 5 min before trials began. Each trial consisted of a 20 ms white noise stimulus (100 dB, 110 dB, or 120 dB) presented from a speaker 20 cm above the mouse’s head. The response of the animal in the 100 ms afterwards was recorded as vibration intensity on the pressure platform (in millivolts, mV); Vavg was the total activity averaged over the recorded window. All three trial tones were repeated five times throughout the experiment, in a pseudorandom order and separated by pseudorandom inter-trial intervals (13–17 s). Trials with significant vibration 100 ms before the tone were excluded from the analysis (<5 instances).

### Electroencephalography (EEG)

#### Surgery

8–10 week old male mice were anesthetized to surgical depth with a ketamine/xylazine mixture (100 mg/kg and 10 mg/kg, respectively). A 2 EEG/1 EMG headmount (8201, Pinnacle Technology) was aligned with the front 3 mm anterior to the bregma and glued to the top of the skull. Four guideholes were hand-drilled, and screws inserted to attach the headmount. The EEG2 channel was used (screws at AP: +1.5 mm, ML: +1.5 mm and AP: −4.0 mm, ML: +1.5 mm to bregma). EMG wires were then inserted into the left and right neck muscles. After skin closing, the headmount was sealed to the skull using dental cement. All animals recovered from surgery for >5 days before being affixed to the EEG recording rigs.

#### EEG recording

Sleep behavioral data were obtained using the Pinnacle Technology EEG/EMG tethered recording system. Following recovery, animals were placed into an 8 in diameter round acrylic cage with lid, provided *ad libitum* food and water, and tethered to a 100x preamplifier. All mice were housed in a 12:12 h LD cycle and acclimated to the cable tethering for ≥5 days prior to recording. EEG and EMG channels were sampled at 400 Hz, high-pass filtered at 0.5 Hz for EEG and 10 Hz for EMG, digitized, and then acquired using Sirena software (Pinnacle Technology). The EEG signal was derived from the posterior “EEG2” screw in combination with the anterior reference screw.

#### Analysis

Mouse sleep was scored visually in Sirenia Sleep software v2.2.7 (Pinnacle Technology) by one or two trained and blinded scorers using raw EEG/EMG traces in 10 s epochs. Each epoch was scored WAKE, NREM, or REM, and epochs with artifacts were marked for exclusion in further analysis. Animals with severe movement artifacts or poor EEG waveforms were excluded from all behavioral datasets. Sleep or wake bouts were identified as >30 s of continuous sleep or wakefulness, respectively. Spectral analysis was performed using custom MATLAB (Mathworks) programs, and all Fast Fourier Transform spectra used 1024 or 512 point size and the Welch’s power spectral density estimate. Spectrograms were composed with short-time Fourier transforms with a window size of 30 s, 60% overlap, and smoothened by a rolling Hann window. To quantify EMG amplitude, wake bouts were extracted from the EMG data using the scored EEG, and relative EMG amplitude was calculated before, during, and after optogenetic activation by normalizing to average EMG amplitude during NREM sleep before optogenetic activation.

### Stereotaxic surgeries

Eight to twelve week old male mice were anesthetized to surgical depth with a ketamine/xylazine mixture (100 mg/kg and 10 mg/kg, respectively). The mouse was secured into a Stoelting stereotaxis frame, and small (~0.5 mm) craniotomies were performed to allow for virus injection (50–300 nl at ~25 nl/min). Coordinates, volumes, and viruses used are listed in Supplementary Table 1. Post-injection, animals were allowed to recover and express viral genes for ≥2 weeks (for anatomical studies), and ≥3 weeks (for all behavior and functional manipulations). If sleep behavior was measured, EEG headmounts were implanted in a separate surgery. Locations of viral injections were confirmed by post-hoc fluorescence imaging.

### Generation and validation of intersectional genetic approaches

To try to isolate glutamatergic vs GABAergic DMH^mWAKE^ neurons, a variety of approaches using Cre and Flp-expressing mouse lines (combined with stereotaxic AAV injections into the DMH) were utilized: *mWake*^*(Cre/+)*^*/Vgat*^*(Flp/+)*^ (AAV-Con/Fon-ChR2 or AAV-Con/Foff-ChR2) or *mWake*^*(Cre/+)*^*/Vglut2*^*(Flp/+)*^ (AAV-Con/Fon-ChR2 or AAV-fDIO-tTA with AAV-TRE-DIO-ChR2) (Supplementary Table 1). To validate these approaches, AAV-Con/Fon-EYFP or AAV-Con/Foff-EFYP was injected into the relevant strain (2–4 month old female mice) and 2–3 weeks later, animals were deeply anesthetized and then perfused with 4% paraformaldehyde in 1x PBS. RNAscope in situ hybridization was then performed using the RNAscope® Multiplex Fluorescent Reagent Kit V2 (Advanced Cell Diagnostics/ACD, 323100). 12 μm sections were used for RNAscope in situ hybridization according to the manufacturer’s protocol (320293). Target probes Vglut2-C2 (319171-C2), Vgat-C2 (319191-C2), tdTomato-C3 (317041-C3) were used. OpalTM Dye (AKOYA Biosciences OpalTM 4-Color Manual IHC Kit, NEL810001KT, 1:2000) was applied to develop the signal (OpalTM 570 and 690). Next, immunostaining was performed using chicken anti-GFP (Invitrogen, A10262, 1:1000), and goat anti-chicken Alexa Fluor 488 (Invitrogen, A-11039; 1:1000) antibodies. ProLong™ Gold Antifade Mountant (Invitrogen, P36930) was immediately placed on each slide, followed by a glass coverslip. Imaging was performed on a Zeiss LSM700 confocal microscope under 40x magnification. Data were analyzed by manually counting the number of total GFP^+^ cells and *Vgat*^*+*^/GFP^+^ or *Vglut2*^*+*^/GFP^+^ cells using Zen Blue (Zeiss) from 3 animals (3–5 slices per animal). For determining whether a cell was *Vgat*^*+*^ or *Vglut2*^*+*^, a threshold of ≥4 RNAscope signal puncta per cell was used.

### Optogenetics

AAVDJ-hSyn-Con/Fon-hChR2(H134R)-EYFP-WPRE or AAV9-EF1a-double floxed-hChR2(H134R)-EYFP-WPRE-HGHpA viruses were stereotaxically injected into the DMH of *mWake*^*(Cre/+)*^*;Vgat*^*(Flp/+)*^ or *mWake*^*(Cre/+)*^ mice, respectively, as described above and in Supplementary Table 1. An optical fiber (200 µm O.D., 0.39 NA, Thorlabs) within a ceramic ferrule was then inserted towards the DMH, and an EEG head mount (Pinnacle Technology) was attached to the skull with 4 screws. Dental acrylic was applied to affix the ferrule and EEG headmount on the skull. Mice were allowed to recover for at least 2 weeks after the surgery. Then, the animals were connected to the optogenetic system (Thorlabs, OGKL2) and the EEG recording rigs (Pinnacle Technology). EEG was recorded after at least 4 days of habituation, and optogenetic stimulation was randomly applied for 10 min between ZT2 to ZT7 (470 nm, 4.5–4.9 mW, 10 Hz, 20% light on). For EEG analyses, 4–9 trials per mouse were recorded over 2–3 days and were only used if the mouse was asleep at the onset of the trial. Video was acquired using a top-mounted camera (DMK 22AUC03, LORE^+^ Technology), sampling at 15 Hz. Locomotor behavior was quantified using ANY-maze software (Stoelting Company), and time spent feeding was quantified manually by an observer. For video analyses, 3–7 trials per mouse were recorded over 1–2 days and were only used if the mouse was asleep at the onset of the trial.

### Electrophysiological recordings

Male mice between 5 and 10 weeks old were deeply anesthetized with isoflurane, and the brains quickly removed and dissected in oxygenated (95% O_2_, 5% CO_2_) ice-cold slicing solution (2.5 mM KCl, 1.25 mM NaH_2_PO_4_, 2 mM MgSO_4_, 2.5 mM CaCl_2_, 248 mM sucrose, 26 mM NaHCO_3_ and 10 mM glucose). Acute coronal brain slices (250 μm) were prepared using a vibratome (VT-1200s, Leica) and then incubated in oxygenated artificial cerebrospinal fluid (ACSF, 124 mM NaCl, 2.5 mM KCl, 1.25 mM NaH_2_PO_4_, 2 mM MgSO_4_, 2.5 mM CaCl_2_, 26 mM NaHCO_3_ and 10 mM glucose, 290–300 mOsm) at 28 °C for 30 min and then at room temperature for 1 h. Slices were then transferred to a recording chamber, continuously perfused with oxygenated ACSF at room temperature and visualized using an upright microscope (BX51WI, Olympus). Labeled cells of interest were visualized using infrared differential interference contrast (IR-DIC) and native fluorescence. Glass electrodes (5–8 MΩ) were filled with the following internal solution (130 mM K-gluconate, 5 mM NaCl, 10 mM C_4_H_8_N_3_O_5_PNa_2_, 1 mM MgCl_2_, 0.2 mM EGTA, 10 mM HEPES, 2 mM MgATP and 0.5 mM Na_2_GTP, pH 7.2–7.3, 300 mOsm). Whole-cell patch clamp recordings were obtained using a Multiclamp 700B amplifier (Molecular Devices). Data were sampled at 20 kHz, low-pass filtered at 2 kHz, and digitized using a Digidata 1440 A (Molecular Devices).

For baseline spontaneous and evoked firing rate measurements, recordings were performed under current clamp configuration. Baseline recordings were performed for at least 30 s to measure spontaneous firing rate. To measure evoked firing rate, current injections from −10 to 100 pA were performed; for each step, current was injected for 100 ms every 2 s. For recordings of DMH^mWAKE^ neurons, ~30% cells exhibited spontaneous firing, and so analyses of evoked responses were restricted to this subset of neurons.

### In vivo fiber photometry

AAV-Flex-GCaMP6s virus was injected into the DMH of *mWake*^*(Cre/+)*^ mice as described above and in Supplementary Table 1. An optical fiber (400 µm O.D., 0.57 NA; Doric Instruments) within a metal ferrule (1.25 mm O.D., Doric Instruments) was then inserted towards the DMH, and an EEG head mount (Pinnacle Technology) was attached to the skull with 4 screws. Dental acrylic was applied to affix the ferrule and EEG headmount on the skull. Mice were allowed to recover at least 14 days after the surgery. GCaMP6s and isosbestic fluorescence were collected from the same implant/patch cable; Ca^2+^-dependent (525 nm) and isosbestic control (430 nm) fluorescence signals (corresponding to 465 nm and 405 nm excitation, respectively) were relayed to the LED driver (Doric Instruments) through dichroic mirrors and bandpass filters within the Fluorescence Mini Cube (Doric Instruments). Doric Neuroscience Studio software (Doric Instruments) was used to control and modulate excitation light (465 nm, 572.205 Hz; 405 nm, 208.616 Hz) under lock-in mode, and fluorescence signals were recorded for 15 min followed by a 15 min break at a 12 KHz sampling rate via the fiber photometry console. Six trials were performed from ZT0-3 or ZT12-15 over 1 or 2 days, and whether recordings began at ZT0 or ZT12 was alternated between different animals. The fiber photometry console sent a 1 s TTL input to the EEG recording system every 3 min for synchronization.

Data were analyzed using custom MATLAB (MathWorks) script. Collected GCaMP, EEG and EMG data were first time-aligned using the TTL pulse sent by the fiber photometry DAQ (Doric Lenses) to the EEG/EMG data acquisition device (Pinnacle Technologies). Bleaching artifacts were removed by low-pass filtering both the reference and GCaMP fluorescence at 0.1 Hz and fitting with an exponential curve. The resulting curve was then subtracted from the reference and GCaMP fluorescence trace. Motion artifacts were corrected by subtracting the bleach-corrected reference signal from GCaMP fluorescence. Z-score was then calculated by subtracting the median from the signal and dividing it by the median absolute deviation. The final Z-score was low pass filtered at 5 Hz. For each ZT time point, six trials (15 min per trial) were averaged to calculate the average fiber photometry trace. These data were then averaged per animal to calculate average Z-scores per state per animal.

Transient peaks were then filtered by using the *findpeaks* function in MATLAB setting the minimum peak width to 80 ms and minimum peak prominence to 0.1. Transient prominence was defined as the height of the signal from the baseline. State transitions for each state were filtered by selecting those with +/−15 s of consolidated states prior to the transition point. The resulting traces were normalized to individual maximums and then plotted as heat maps.

### Designer Receptors Exclusively Activated by Designer Drugs (DREADDs)

DREADD receptors coupled to either Gq or Gi were expressed in a Cre-dependent fashion in *mWake*^*+*^ neurons of *mWake*^*(Cre/+)*^ mice via stereotaxic injection of a viral vector (AAV-DIO-hM3D-Gq or AAV-DIO-hM4D-Gi) (Supplementary Table 1). Clozapine *N*-oxide (CNO) (SigmaAldrich) was prepared as a stock solution of 50 mg/ml in DMSO, and then freshly diluted to 0.1 mg/ml in sterile PBS before IP injection. Solution clarity was monitored throughout dosing, and the solution was warmed to 37 °C if precipitates were observed. Vehicle control was prepared as sterile saline + 0.01% DMSO. All injections occurred at the same ZT/CT time within each experiment, and all animals were treated with vehicle or CNO each day in a cross-over design, with ≥2 days recovery between experimental recording days. To control for CNO activity on its own, 1 or 3 mg/kg CNO were IP injected into sham-injected *mWake*^*(Cre/+)*^ mice and locomotion and EEG data were assessed.

### Statistical analysis

Statistical analyses were performed in Prism 7 and 8 (GraphPad). For comparisons of two groups of normally distributed data, unpaired Welch’s t-tests were used; if these comparisons were before and after treatment of the same animals or cells, paired t-tests were used instead. For comparisons of two groups of non-normally distributed data, Mann Whitney U tests were performed. Holm-Bonferroni corrections were used if required for multiple comparisons. For multiple comparisons of normally distributed data, one-way ANOVAs were performed with post-hoc Tukey or post-hoc Dunnett tests. For multiple comparisons of normally distributed data with 2 factors, two-way ANOVAs were performed (with repeated measures, if applicable), followed by post-hoc Sidak tests. For multiple comparisons of non-normally distributed data, Kruskal–Wallis tests were performed with post-hoc Dunn tests. Data that were not normally distributed were plotted as simplified boxplots (median with 1st and 3rd quartile boxes).

### Reporting summary

Further information on research design is available in the Nature Portfolio Reporting Summary linked to this article.

### Supplementary information

Supplementary Information

Description of Additional Supplementary Files

Supplementary Movie 1

Supplementary Movie 2

Supplementary Movie 3

Reporting Summary

### Source data

Source Data

## Data Availability

The data generated in this study are provided in the Supplementary Information/Source Data file. scRNA-seq data from this study are accessible through GEO Series accession number GSE146166. Source data are provided with this paper.

## Code availability

Analysis scripts for in vivo fiber photometry can be found at https://zenodo.org/record/8298898.
