# Supplementary material for: A clock-dependent brake for rhythmic arousal in the dorsomedial hypothalamus
Source: Nat Commun. 2023 Oct 11;14:6381. doi: 10.1038/s41467-023-41877-4 (PMC10567910; doi:10.1038/s41467-023-41877-4)
Supplement: Supplementary file 1 — Supplementary Information [file 41467_2023_41877_MOESM1_ESM.pdf]

Inventory of Supporting Information

A Clock-Dependent Brake for Rhythmic Arousal in the Dorsomedial Hypothalamus

Qiang Liu*, Benjamin J. Bell*, Dong Won Kim, Sang Soo Lee, Mehmet F. Keles, Qili Liu, Ian D. Blum, Annette A. Wang, Elijah J. Blank, Jiali Xiong, Joseph L. Bedont, Anna J. Chang, Habon Issa, Jeremiah Y. Cohen, Seth Blackshaw, and Mark N. Wu

Correspondence to: marknwu@jhmi.edu

This file includes:

1. Supplementary Fig. 1. mWAKE$^+$ neurons in the DMH
2. Supplementary Fig. 2. mWAKE$^+$ DMH neurons promote arousal and REM sleep
3. Supplementary Fig. 3. Additional data related to *mWake*$^+$ cell identity
4. Supplementary Fig. 4. Validation of intersectional approaches
5. Supplementary Fig. 5. Additional Behavioral Data Related to Figure 4
6. Supplementary Fig. 6. Projection analyses for DMH^{mWAKE} vs GABAergic DMH^{mWAKE} neurons
7. Supplementary Fig. 7. EEG-related phenotypes of *mWake* mutant mice
8. Supplementary Fig. 8. *mWake* mutants are hyperactive at night
9. Supplementary Fig. 9. Additional data related to Fig. 6
10. Supplementary Table 1. Stereotaxic coordinates and viruses injected
11. Supplementary Table 2. Additional electrophysiological properties for DMH^{mWAKE} neurons.
12. Supplementary References

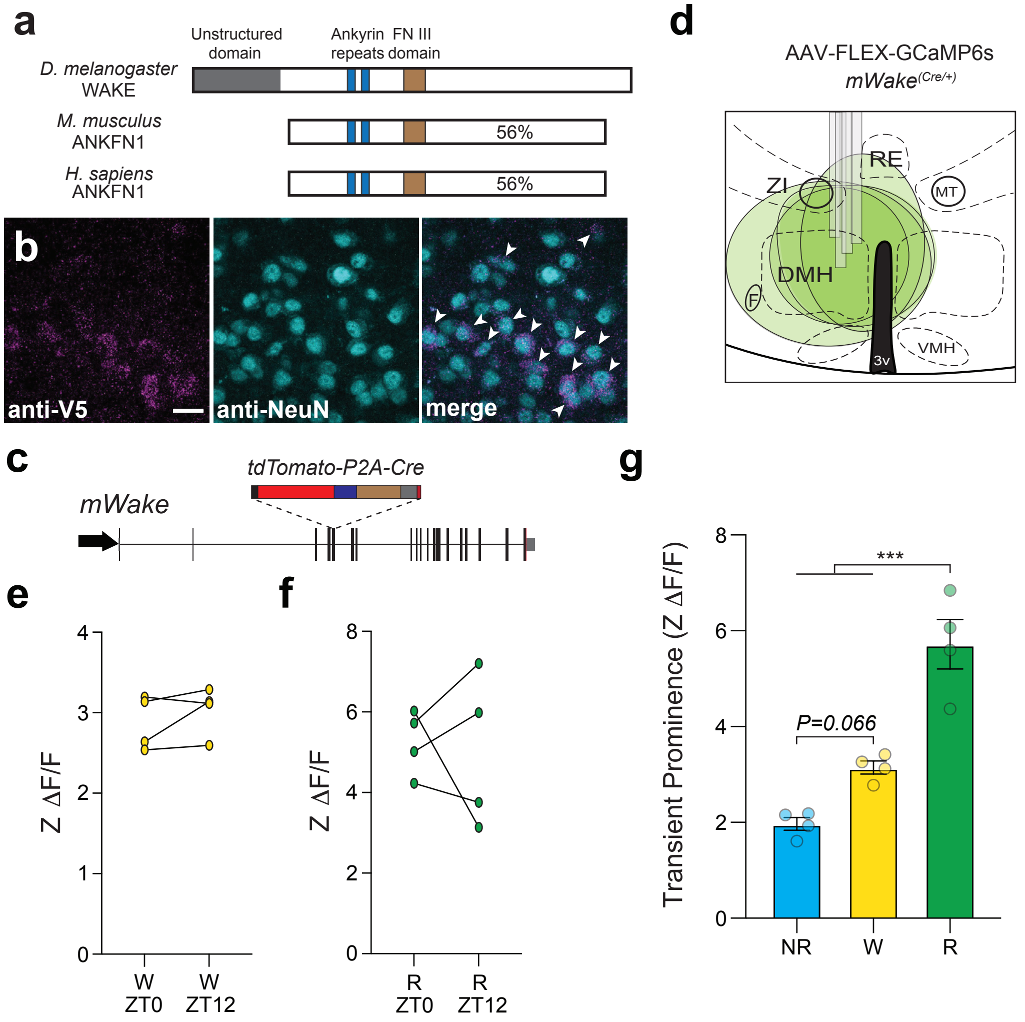

Supplementary Fig. 1. mWAKE^{+} neurons in the DMH

a, Schematic showing domain structure (unstructured domain, ankyrin repeats, and fibronectin type III domain) of mWAKE in mice and humans, compared to *Drosophila* WAKE. Percentage similarity (calculated using the BLOSUM62 comparison matrix) of mouse and human mWAKE, compared to fly WAKE is shown.
b, Confocal images of anti-V5 (left) or anti-NeuN (middle) immunostaining, or merged signals (right) of the DMH from *mWake*$^{(V5/V5)}$ mice. Arrowheads depict DMH^{mWAKE} neurons. Scale bar denotes 20 μm and applies to all panels.
c, Schematic showing genomic structure of the *mWake* locus and replacement of exon 5 with a tdTomato-P2A-Cre-stop cassette in the *mWake*$^{(Cre)}$ line.
d, Schematic adapted from Paxinos and Franklin[51] coronal plate demonstrating extent of viral gene expression (green ovals) and placement of fiber photometry probes in *mWake*$^{(Cre/+)}$ mice injected with AAV-Flex-GCaMP6s. 3v, third ventricle; DMH, dorsomedial hypothalamus; f, fornix; MT, mammillothalamic tract; RE, thalamic reuniens nucleus; VMH, ventromedial hypothalamus; ZI, zona incerta
e, Z-score of ΔF/F signal for wake state compared between ZT0-3 and ZT12-15; two-tailed paired t-test with Holm-Bonferroni correction. Data for **e**-**g** are from the same animals as in Fig. 1.
f, Z-score of ΔF/F signal for REM state compared between ZT0-3 and ZT12-15; two-tailed paired t-test with Holm-Bonferroni correction.
g, Mean transient prominence (Z-score of ΔF/F signal) across sleep-wake stages; one-way ANOVA with post-hoc Tukey, ****P*<0.0001 (NR-R), ****P*=0.0008 (W-R), *P*=0.066 (NR-W). Error bars, SEM.

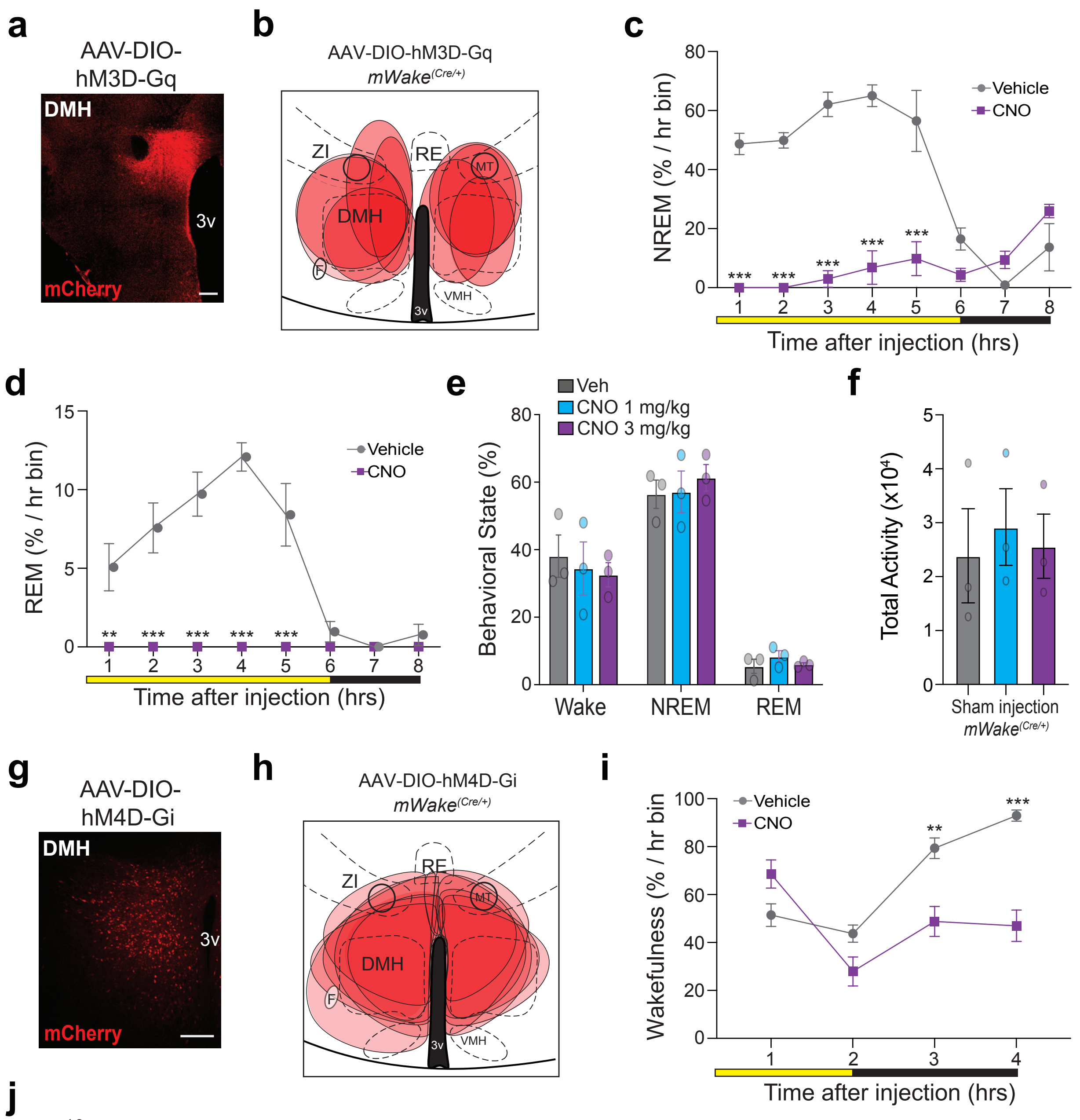

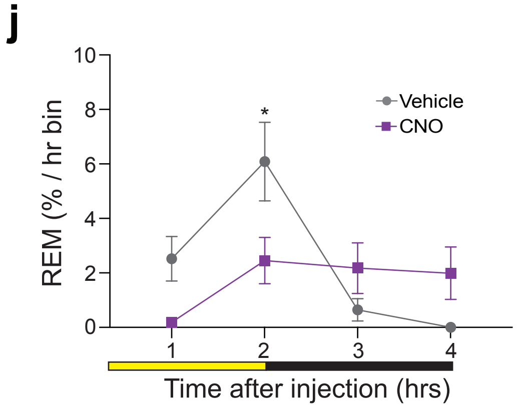

Supplementary Fig. 2. mWAKE$^+$ DMH neurons promote arousal and REM sleep
a, Representative image of mCherry fluorescence expression in the DMH of *mWake*$^{(Cre/+)}$ following AAV-DIO-hM3D-Gq injection described in Fig. 2a. Scale bar denotes 200 µm. "3v" denotes third ventricle.
b, Schematic adapted from Paxinos and Franklin[51] coronal plate demonstrating extent of viral gene expression (red ovals) in *mWake*$^{(Cre/+)}$ mice injected with AAV-DIO-hM3D-Gq.
c and **d**, Amount of NREM (**c**) or REM (**d**) plotted as % time in 1 hr bins for the mice in Fig. 2a, following IP injection of vehicle (gray) or 1 mg/kg CNO (magenta) (n=4) at ZT6; two-way ANOVA with post-hoc Holm-Sidak, ***P<0.0001 (NREM, 1 hrs), ***P<0.0001 (NREM, 2 hrs), ***P<0.0001 (NREM, 3 hrs), ***P<0.0001 (NREM, 4 hrs), ***P<0.0001 (NREM, 5 hrs), **P=0.0014 (REM, 1 hrs), ***P<0.0001 (REM, 2 hrs), ***P<0.0001 (REM, 3 hrs), ***P<0.0001 (REM, 4 hrs), ***P<0.0001 (REM, 5 hrs). Note that these data are from the same mice as in Fig. 2, b-d.
e, Behavioral state (%) of 2 hrs of recorded EEG activity, starting after IP injection of vehicle alone (n=3), 1 mg/kg CNO (n=3), or 3 mg/kg CNO (n=3) at ZT6, from *mWake*$^{(Cre/+)}$ mice with sham injections into the DMH; one-way ANOVA with post-hoc Tukey, followed by Holm-Bonferroni correction.
f, Total locomotor activity for the mice described in (**e**) in the 4 hrs following IP injection of vehicle (gray), CNO (1 mg/kg, cyan), or CNO (3 mg/kg, magenta) (n=3) at ZT6; one-way ANOVA with post-hoc Tukey.
g, Representative image of mCherry fluorescence expression in the DMH of *mWake*$^{(Cre/+)}$ following AAV-DIO-hM4D-Gi injection described in Fig. 2e. Scale bar denotes 200 µm. "3v" denotes third ventricle.
h, Schematic adapted from Paxinos and Franklin[51] coronal plate demonstrating extent of viral gene expression (red ovals) in *mWake*$^{(Cre/+)}$ mice injected with AAV-DIO-hM4D-Gi.
i and **j**, Wakefulness (**i**) and REM (**j**) amount for the mice in Fig. 2e, plotted as % time in 1 hr bins following IP injection of vehicle (gray) or 3 mg/kg CNO (magenta) (n=6) at ZT10; two-way ANOVA with post-hoc Holm-Sidak, **P=0.0022 (wakefulness, 3 hrs), ***P<0.0001 (wakefulness, 4 hrs), *P=0.0186 (REM, 2 hrs). Error bars, SEM.

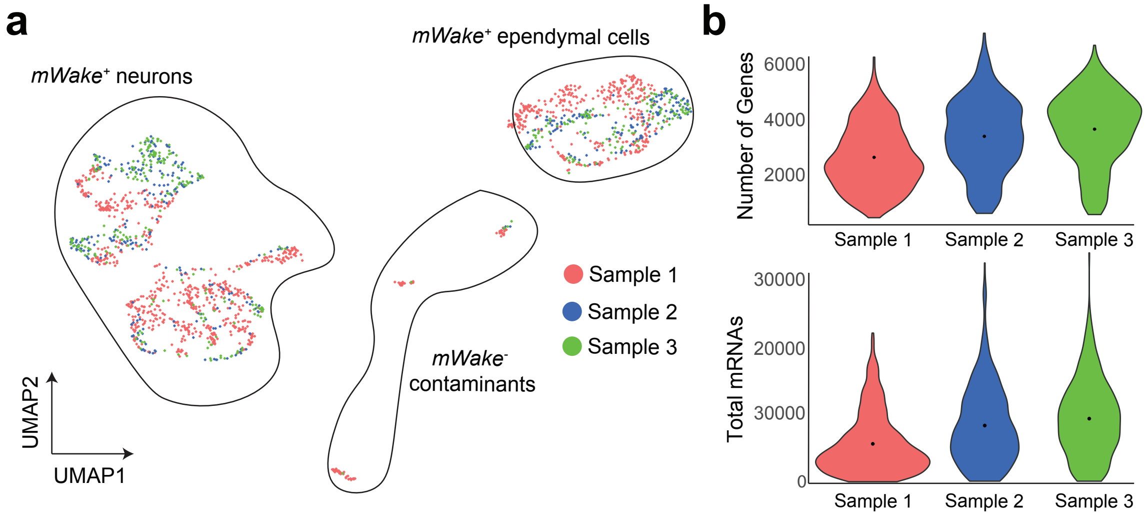

Supplementary Fig. 3. Additional data related to mWake⁺ cell identity

a, UMAP plot showing distribution of mWake⁺ cells in individual scRNA-Seq libraries and distribution of mWake⁺ neurons and ependymal cells.

b, Violin plot showing distribution of number and mean (black dot) of genes (top) and total mRNAs (bottom, calculated by the number of unique molecular identifiers (UMIs)) in individual scRNA-Seq libraries.

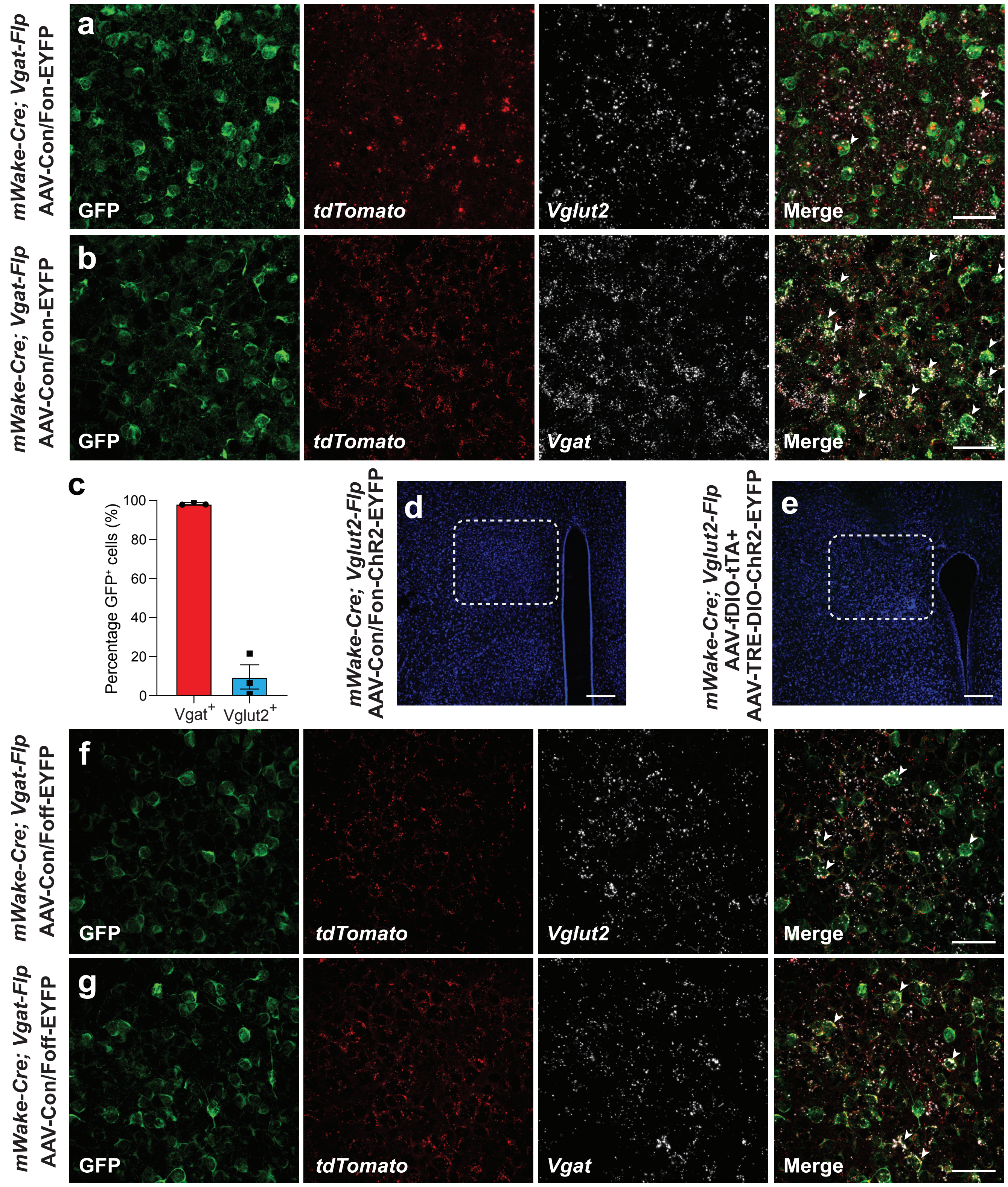

Supplementary Fig. 4. Validation of intersectional approaches

a and **b**, Representative confocal images of the DMH region from *mWake*$^{(Cre/+)}$; *Vgat*$^{(Flp/+)}$ male or female mice injected with AAV-Con/Fon-EYFP. *TdTomato* and *Vglut2* RNAscope probes were used to identify mWAKE$^+$ and Vglut2$^+$ cells, respectively in (**a**), while *TdTomato* and *Vgat* RNAscope probes were used to identify mWAKE$^+$ and Vgat$^+$ cells, respectively in (**b**). anti-GFP immunostaining was used to identify virally-infected cells, and merged images are also shown. Arrowheads indicate representative cells that are mWAKE$^+$ and Vglut2$^+$ or Vgat$^+$, respectively. Scale bar denotes 50 µm.

c, Quantification of % colocalization of *Vgat* and GFP signal vs *Vglut2* and GFP signal from the mice in (**a**) and (**b**). n=3 replicates

d and **e**, Representative confocal images of native EYFP fluorescence from the DMH region from *mWake*$^{(Cre/+)}$; *Vglut2*$^{(Flp/+)}$ male mice injected with AAV-Con/Fon-ChR2-EYFP (**d**) or AAV-fDIO-tTA+AAV-TRE-DIO-ChR2-EYFP (**e**). Scale bar denotes 100 µm. n=3 replicates.

f and **g**, Representative confocal images of the DMH region from *mWake*$^{(Cre/+)}$; *Vgat*$^{(Flp/+)}$ male or female mice injected with AAV-Con/Foff-EYFP. *TdTomato* and *Vglut2* RNAscope probes were used to identify mWAKE$^+$ and Vglut2$^+$ cells, respectively in (**f**), while *TdTomato* and *Vgat* RNAscope probes were used to identify mWAKE$^+$ and Vgat$^+$ cells, respectively in (**g**). anti-GFP immunostaining was used to identify virally-infected cells, and merged images are also shown. Arrowheads indicate representative cells that are mWAKE$^+$ and Vglut2$^+$ or Vgat$^+$, respectively. Scale bar denotes 50 µm. Error bars, SEM. n=3 replicates.

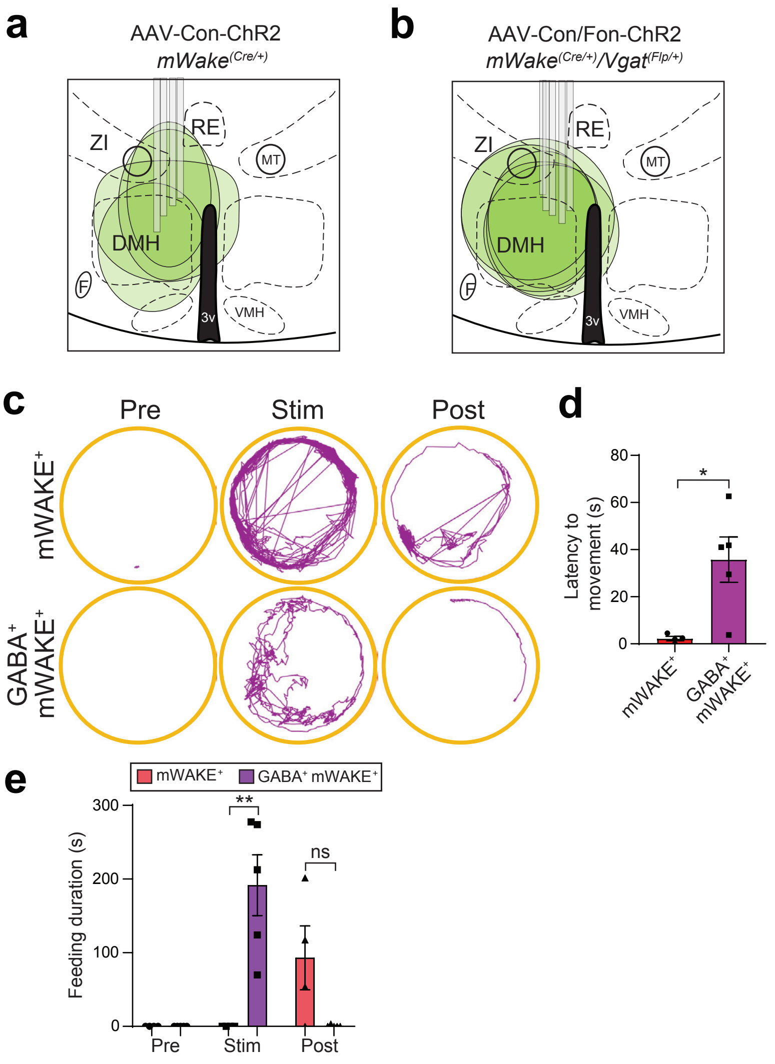

Supplementary Fig. 5. Additional Behavioral Data Related to Figure 4

a and **b**, Schematics adapted from Paxinos and Franklin[51] coronal plates demonstrating extent of viral gene expression (green ovals) and placement of optic fiber in *mWake$^{(Cre/+)}$* mice injected with AAV-Con-ChR2 (**a**) or *mWake$^{(Cre/+)}$*; *Vgat$^{(Flp/+)}$* mice injected with AAV-Con/Fon-ChR2 (**b**).

c, Representative locomotor activity tracks for the 10 min periods before ("Pre"), during ("Stim"), and after ("Post") optogenetic activation of DMH^{mWAKE} neurons or GABAergic DMH^{mWAKE} neurons.

d, Latency to movement following optogenetic activation of DMH^{mWAKE} neurons (red, n=4 animals) or GABAergic DMH^{mWAKE} neurons (magenta, n=5 animals); unpaired t-test, *P=0.0182, two-tailed.

e, Feeding duration for the 10 min period before, during, or after optogenetic stimulation of the DMH^{mWAKE} neurons (red, n=4 animals) or GABAergic DMH^{mWAKE} neurons (magenta, n=5 animals); two-way ANOVA with post-hoc Sidak, **P=0.0011 (Stim), P=0.0745 (Post). Data in (**d**) and (**e**) were analyzed from the same mice as in Figs. 4j and 4k. Error bars, SEM.

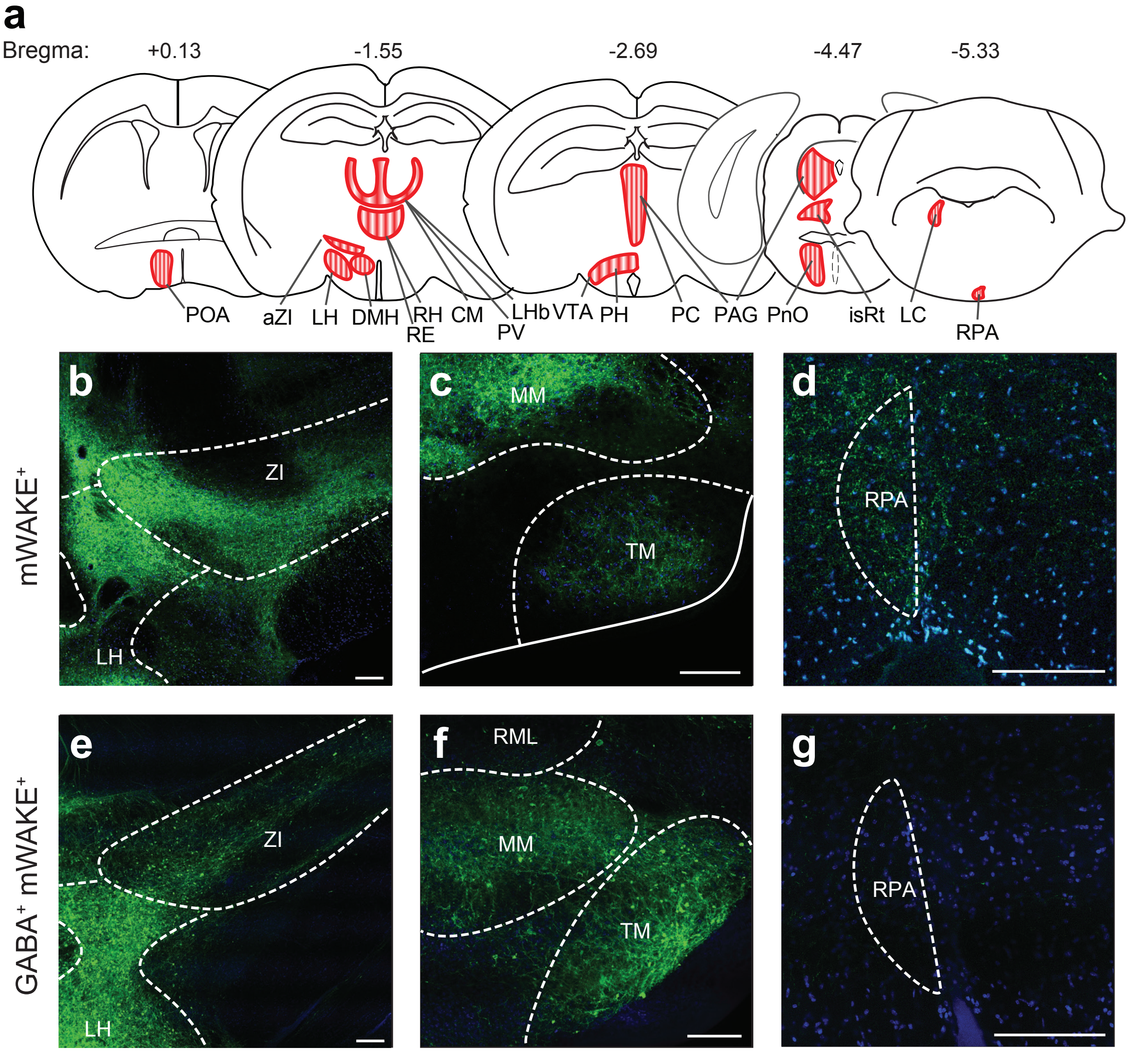

Supplementary Fig. 6. Projection analyses for DMH^{mWAKE} vs GABAergic DMH^{mWAKE} neurons

a, Schematics adapted from Paxinos and Franklin[51] coronal plates depicting putative projection targets of DMH^{mWAKE} neurons. A-P coordinates relative to bregma are shown. aZI, anterior zona incerta; CM, central medial thalamic nucleus; DMH, dorsomedial hypothalamus; isRT, isthmic reticular formation; LC, locus coeruleus; LH, lateral hypothalamus; LHb, lateral habenula; PAG, periaqueductal gray; PC, posterior commissure; PH, posterior hypothalamus; PnO, pontine reticular nucleus (oral); POA, preoptic area; PV, paraventricular thalamic nucleus; RE, nucleus reuniens; RH, rhomboid thalamic nucleus; RPA, raphe pallidus nucleus; VTA, ventral tegmental area.

b-**g**, Representative confocal images showing ChR2-EYFP signal for DMH^{mWAKE} neurons (**b**-**d**) and GABAergic DMH^{mWAKE} neurons (**e**-**g**) and also DAPI signal. LH, lateral hypothalamus; MM, medial mammillary; RPA, raphe pallidus nucleus; TM, tuberomammillary; ZI, zona incerta;. Scale bars in (**b**-**g**) denote 150 mm.

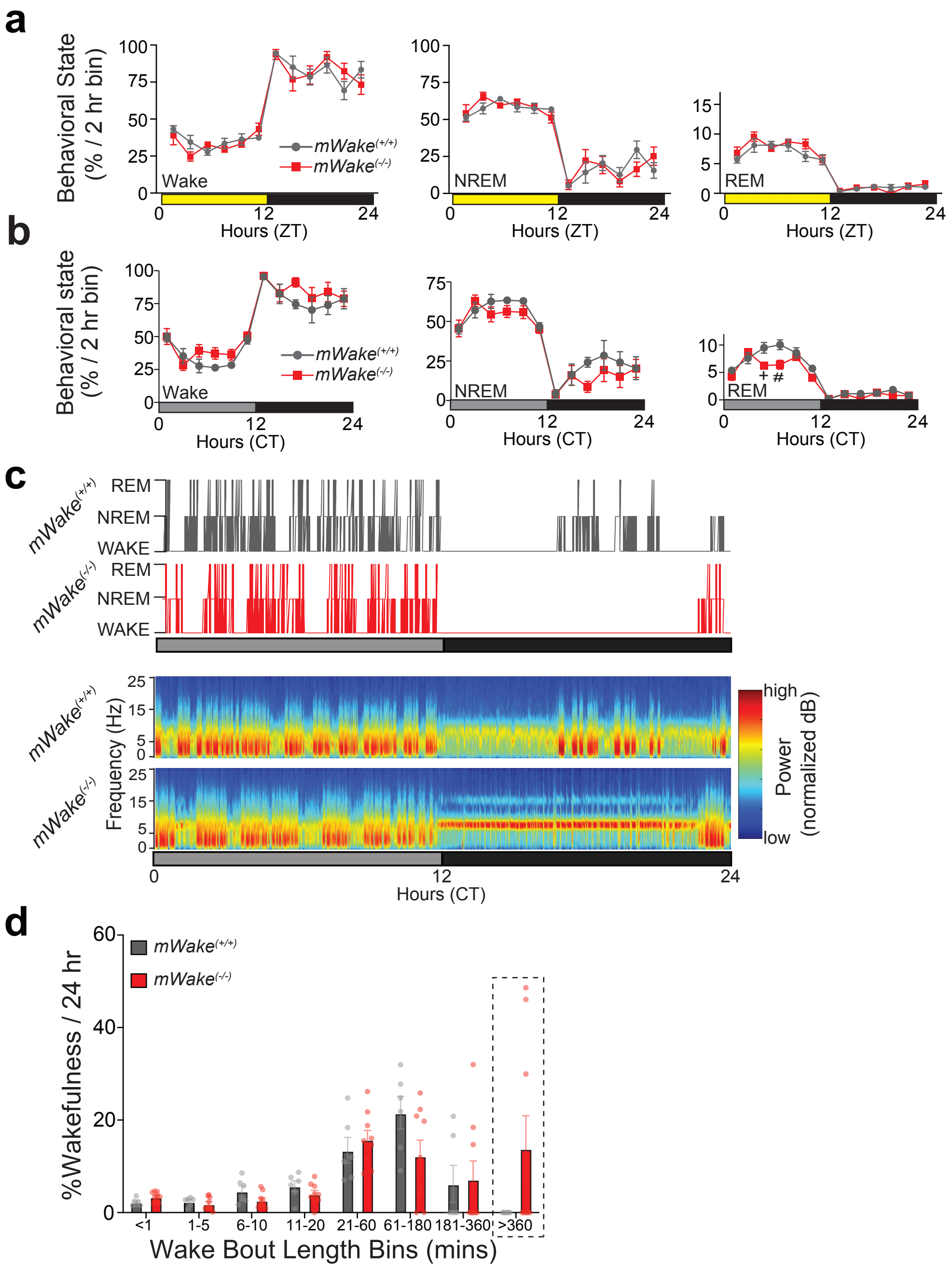

Supplementary Fig. 7. EEG-related phenotypes of *mWake* mutant mice

a and **b**, Behavioral state (% per 2 hr bin) determined by EEG recordings for *mWake*$^{(-/-)}$ vs WT littermate control mice under LD (n=8 vs n=7, respectively) (**a**) or DD (n=8 vs n=6, respectively) (**b**) conditions; two-way ANOVA with post-hoc Holm-Sidak, "+" and "#" denote ***P*=0.0022 and ****P*=0.0004, respectively. n=3 replicates.

c, Hypnograms (top) and short-time Fourier transform spectrograms (bottom) over 24 hrs in DD showing example of prolonged wake bout in a *mWake*$^{(-/-)}$ mouse, compared to a *mWake*$^{(+/+)}$ control. Power density is represented by the color-scheme and deconvoluted by frequency on the y-axis, over time on the x-axis.

d, Wakefulness bout length distribution over a 24 hr period expressed as a % of total wakefulness, for *mWake*$^{(+/+)}$ (n=6) vs *mWake*$^{(-/-)}$ (n=9) mice. Dashed line highlights the presence of >6 hr continuous bouts of wakefulness, which are observed in some *mWake*$^{(-/-)}$ mutants and never in controls. Error bars, SEM. n=3 replicates.

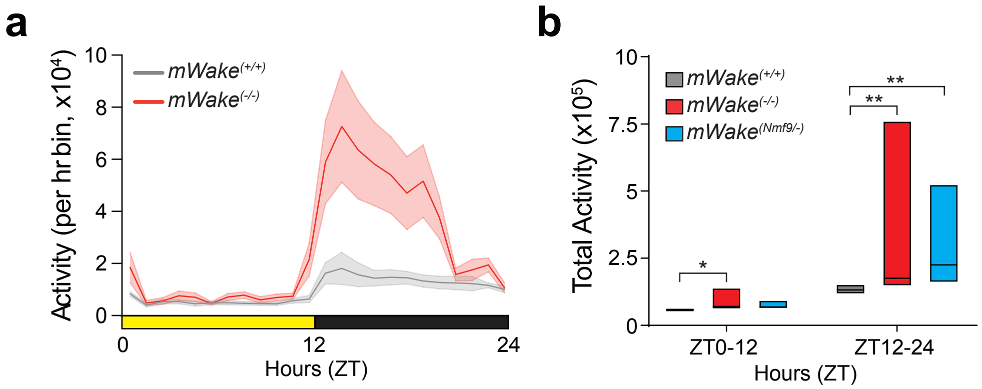

Supplementary Fig. 8. *mWake* mutants are hyperactive at night

a, Profile of locomotor activity (defined by beam breaks) over 24 hrs for *mWake*$^{(+/+)}$ (gray) and *mWake*$^{(-/-)}$ (red) mice under LD conditions. Shading denotes SEM.

b, Total locomotor activity for *mWake*$^{(+/+)}$ (n=19, gray), *mWake*$^{(-/-)}$ (n=19, red), and *mWake*$^{(Nmf9/-)}$ (n=10, cyan) mice at ZT0-12 and ZT12-24; Kruskal-Wallis test with post-hoc Dunn, **P*=0.0408 (ZT0-12: *mWake*$^{(+/+)}$-*mWake*$^{(-/-)}$), ***P*=0.0015 (ZT12-24: *mWake*$^{(+/+)}$-*mWake*$^{(-/-)}$), ***P*=0.0038 (ZT12-24: *mWake*$^{(+/+)}$-*mWake*$^{(Nmf9/-)}$). Simplified boxplots show 25th percentile, median, and 75th percentile. Note that these data are from the same mice as in Fig. 5h.

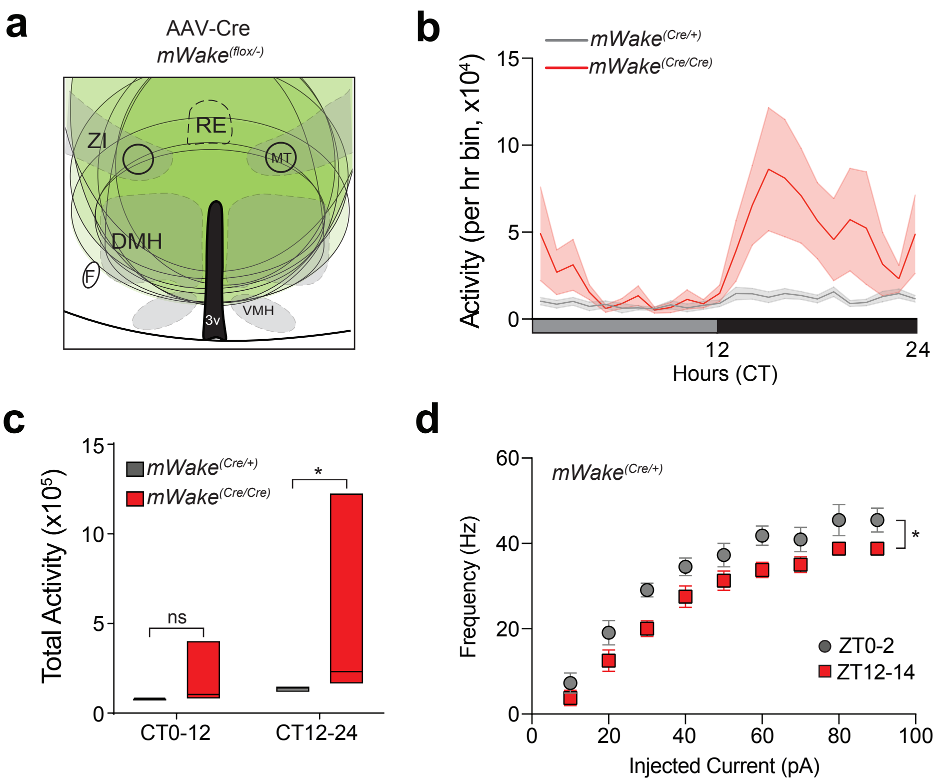

Supplementary Fig. 9. Additional data related to Fig. 6

a, Schematic adapted from Paxinos and Franklin[51] coronal plate demonstrating extent of viral gene expression (green ovals) in *mWake*$^{(flox/-)}$ mice injected with AAV-Cre-GFP. Gray shading indicates regions with mWAKE expression[21].

b, Locomotor activity profile over 24 hrs for *mWake*$^{(Cre/+)}$ (gray) and *mWake*$^{(Cre/Cre)}$ (red) under DD conditions. Shading denotes SEM.

c, Total locomotor activity for *mWake*$^{(Cre/+)}$ (n=9, gray) and *mWake*$^{(Cre/Cre)}$ (n=10, red) at CT0-12 vs CT12-24; Mann-Whitney U test with Holm-Bonferroni correction, *P*=0.1564 (CT0-12), **P*=0.0114 (CT12-24), two-tailed. Simplified boxplots show 25th percentile, median, and 75th percentile. n=3 replicates.

d, f-I curves for DMH^{mWAKE} neurons from *mWake*$^{(Cre/+)}$ mice at ZT0-2 (gray) vs ZT12-14 (red); two-way ANOVA with repeated measures, **P*=0.021. This plot derives from the same dataset as shown in Fig. 6h. Error bars, SEM.

Supplementary Table 1. Stereotaxic coordinates and viruses injected

Location	Coordinates	Virus (Source)	Use	Vol.	Laterality
**DMH**	AP:-1.55, ML:+/-0.25, DV:-5.55	AAV9.CMV.HI.eGFP-Cre.WPRE.SV40 (Penn Vector) "AAV-Cre"	Conditional KO	300 nl	Bi-
	AP:-1.55, ML:+/-0.25, DV:-5.55	AAV9.CMV.HI.eGFP-WPRE.SV40 (Penn Vector) "AAV-Sham"	Sham-Control	300 nl	Bi-
	AP:-1.55, ML:+/-0.25, DV:-5.55	AAV9.hSyn.DIO.hM3D(Gq)-mCherry (Addgene# 44361) "AAV-DIO-hM3D-Gq"	DREADD activation	250 nl	Bi-
	AP:-1.55, ML:+/-0.42, DV:-5.0	AAV8.hSyn.DIO.hM4D(Gi)-mCherry (Addgene# 44362) "AAV-DIO-hM4D-Gi"	DREADD inhibition	250 nl	Bi-
	AP:-1.55, ML:+0.42, DV:-5.0	AAV9.Syn.Flex.GCaMP6s.WPRE.SV40 (Addgene# 100845) "AAV-DIO-GCaMP6s"	Fiber Photometry	300 nl	Uni-
	AP:-1.55, ML:+0.42, DV:-5.0	AAVDJ-hSyn-Con/Fon hChR2 (H134R)-EYFP-WPRE (UNC Vector Core) "AAV-Con/Fon-ChR2"	Intersectional	300 nl	Uni-
	AP:-1.55, ML:+0.42, DV:-5.0	AAV9-EF1a-double floxed-hChR2 (H134R)-EYFP-WPRE-HGHpA (Addgene: 20298) "AAV-Con-ChR2"	Intersectional	300 nl	Uni-
	AP:-1.55, ML:+0.42, DV:-5.0	AAVDJ-hSyn-Con/Foff hChR2 (H134R)-EYFP-WPRE (UNC Vector Core) "AAV-Con/Foff-ChR2"	Intersectional	300 nl	Uni-
	AP:-1.55, ML:+0.42, DV:-5.0	AAV-hSYN1-fDIO-tTA (Addgene: 166597) Packaged by Biohippo "AAV-fDIO-tTA"	Intersectional	300 nl	Uni-
	AP:-1.55, ML:+0.42, DV:-5.0	AAV-TRE-DIO-ChR2-EYFP, modified from "AAV-TRE-DIO-ChR2-TdTomato (Addgene: 166610) Packaged by Biohippo "AAV-TRE-DIO-ChR2"	Intersectional	300 nl	Uni-
	AP:-1.55, ML:+0.42, DV:-5.0	AAV8-hSyn-Con/Fon-EYFP (Addgene# 55650) "AAV-Con/Fon-EYFP"	Intersectional	200 nl	Bi-
	AP:-1.55, ML:+0.42, DV:-5.0	AAVDJ-hSyn-Con/Foff-EYFP (UNC Vector Core) "AAV-Con/Foff-EYFP"	Intersectional	200 nl	Bi-

Genotype and condition	MFR (Hz)	RMP (mV)	Rin (GΩ)	τ (ms)
mWAKE$^{Cre/+}$ DMH ZT0-2	0.6 ± 1.0	-54.6 ± 3.2	1.3 ± 0.1	63.1 ± 0.9
mWAKE$^{Cre/Cre}$ DMH ZT0-2	0.74 ± 0.2<br>(*P*=0.43)	-56.4 ± 3.8<br>(*P*=0.72)	1.6 ± 0.1<br>(*P*=0.11)	64.3 ± 0.2<br>(*P*=0.22)
mWAKE$^{Cre/+}$ DMH ZT12-14	1.2 ± 0.2	-53.2 ± 1.8	1.5 ± 0.1	63.2± 0.1
mWAKE$^{Cre/Cre}$ DMH ZT12-14	2.2 ± 0.5<br>(*P*=0.01)	-47.7 ± 2.0<br>(*P*=0.06)	1.4 ± 0.2<br>(*P*=0.82)	63.2 ± 0.4<br>(*P*=0.92)

Supplementary Table 2. Additional electrophysiological properties for mWake^{DMH} neurons. Mean firing rate (MFR), resting membrane potential (RMP), input resistance (Rin), and membrane time constant (τ) are shown for the displayed genotypes and cells during ZT0-2 and ZT12-14. *P* values are shown for comparisons between ZT0-2 and ZT12-14 for a given genotype.

Supplementary References

51. Paxinos, G. & Franklin, K. The Mouse Brain in Stereotaxic Coordinates (4th ed.). *Acad. Press San Diego* (2012).
