# Supplementary material for: A clock-dependent brake for rhythmic arousal in the dorsomedial hypothalamus
Source: Nat Commun. 2023 Oct 11;14:6381. doi: 10.1038/s41467-023-41877-4 (PMC10567910; doi:10.1038/s41467-023-41877-4)
Supplement: Supplementary file 2 — Description of Additional Supplementary Files [file 41467_2023_41877_MOESM2_ESM.pdf]

Description of Additional Supplementary Files

File Name: Supplementary Movie 1
Description: Optogenetic activation of DMH^{mWAKE} neurons

File Name: Supplementary Movie 2
Description: Optogenetic activation of GABAergic DMH^{mWAKE} neurons

File Name: Supplementary Movie 3
Description: *mWake* KO mice exhibit nighttime hyperactivity and circling.
