# Supplementary material for: A clock-dependent brake for rhythmic arousal in the dorsomedial hypothalamus
Source: Nat Commun. 2023 Oct 11;14:6381. doi: 10.1038/s41467-023-41877-4 (PMC10567910; doi:10.1038/s41467-023-41877-4)
Supplement: Supplementary file 6 — Reporting Summary [file 41467_2023_41877_MOESM6_ESM.pdf]

nature portfolio

Corresponding author(s): Wu, MN

Last updated by author(s): Sep 7, 2023

Reporting Summary

Nature Portfolio wishes to improve the reproducibility of the work that we publish. This form provides structure for consistency and transparency in reporting. For further information on Nature Portfolio policies, see our Editorial Policies and the Editorial Policy Checklist.

Statistics

For all statistical analyses, confirm that the following items are present in the figure legend, table legend, main text, or Methods section.

n/a	Confirmed	
☐	☒	The exact sample size (*n*) for each experimental group/condition, given as a discrete number and unit of measurement
☐	☒	A statement on whether measurements were taken from distinct samples or whether the same sample was measured repeatedly
☐	☒	The statistical test(s) used AND whether they are one- or two-sided<br>*Only common tests should be described solely by name; describe more complex techniques in the Methods section.*
☒	☐	A description of all covariates tested
☐	☒	A description of any assumptions or corrections, such as tests of normality and adjustment for multiple comparisons
☐	☒	A full description of the statistical parameters including central tendency (e.g. means) or other basic estimates (e.g. regression coefficient) AND variation (e.g. standard deviation) or associated estimates of uncertainty (e.g. confidence intervals)
☐	☒	For null hypothesis testing, the test statistic (e.g. *F*, *t*, *r*) with confidence intervals, effect sizes, degrees of freedom and *P* value noted<br>*Give P values as exact values whenever suitable.*
☒	☐	For Bayesian analysis, information on the choice of priors and Markov chain Monte Carlo settings
☒	☐	For hierarchical and complex designs, identification of the appropriate level for tests and full reporting of outcomes
☒	☐	Estimates of effect sizes (e.g. Cohen's *d*, Pearson's *r*), indicating how they were calculated

Our web collection on statistics for biologists contains articles on many of the points above.

Software and code

Policy information about availability of computer code

Data collection	Oxymax software v5.27 (Columbus Instruments) was used to collect locomotor activity data. Sirenia Sleep software v2.2.7 (Pinnacle Technology) was used to collect EEG/sleep data.
Data analysis	Imaris 9.5 software (Oxford Instruments) was used to quantify neuron numbers. Cellranger 3.1 (10x Genomics) and Seurat V3 were used for scRNA-seq analyses. Custom MATLAB code was used to analyze fiber photometry data (https://zenodo.org/record/8298898). Statistical analyses were performed using Prism 7 and Prism 8.

For manuscripts utilizing custom algorithms or software that are central to the research but not yet described in published literature, software must be made available to editors and reviewers. We strongly encourage code deposition in a community repository (e.g. GitHub). See the Nature Portfolio guidelines for submitting code & software for further information.

Data

Policy information about availability of data

All manuscripts must include a data availability statement. This statement should provide the following information, where applicable:

- Accession codes, unique identifiers, or web links for publicly available datasets
- A description of any restrictions on data availability
- For clinical datasets or third party data, please ensure that the statement adheres to our policy

The data generated in this study are provided in the Supplementary Information/Source Data file. scRNA-seq data from this study are accessible through GEO Series accession number GSE146166 (https://www.ncbi.nlm.nih.giv/geo/query/acc.cgi?acc=gse146166)

Research involving human participants, their data, or biological material

Policy information about studies with human participants or human data. See also policy information about sex, gender (identity/presentation), and sexual orientation and race, ethnicity and racism.

Reporting on sex and gender	N/A
Reporting on race, ethnicity, or other socially relevant groupings	N/A
Population characteristics	N/A
Recruitment	N/A
Ethics oversight	N/A

Note that full information on the approval of the study protocol must also be provided in the manuscript.

Field-specific reporting

Please select the one below that is the best fit for your research. If you are not sure, read the appropriate sections before making your selection.

☒ Life sciences ☐ Behavioural & social sciences ☐ Ecological, evolutionary & environmental sciences

For a reference copy of the document with all sections, see nature.com/documents/nr-reporting-summary-flat.pdf

Life sciences study design

All studies must disclose on these points even when the disclosure is negative.

Sample size	Sample size was not calculated a priori, as mWAKE is a novel gene and labels a novel circuit. We thus used sample sizes that were in line with those typically used for a given experiment (behavioral, EEG/EMG, in vivo Ca2+ imaging, patch-clamp recordings).
Data exclusions	No data were excluded
Replication	The number of biological replicates is provided in the figure legends.
Randomization	Mice were randomly assigned to control and experimental groups, and if a paired analysis was performed, the ordering of control vs experimental conditions were also randomized and balanced.
Blinding	Experimenters were blinded to animal genotype (including EEG analyses), except for patch-clamp recordings, where the experimenter had to dissect an animal of a specific genotype and experimenter bias has little impact on the data. Experimenters were not blinded to chemogenetic or optogenetic manipulations, because the experimenter had to prepare the appropriate solution or light stimulation protocol. In addition, objective (not subjective) readouts were used for chemogenetic and optogenetic manipulations.

Reporting for specific materials, systems and methods

We require information from authors about some types of materials, experimental systems and methods used in many studies. Here, indicate whether each material, system or method listed is relevant to your study. If you are not sure if a list item applies to your research, read the appropriate section before selecting a response.

Materials & experimental systems

n/a	Involved in the study
☐	☒ Antibodies
☒	☐ Eukaryotic cell lines
☒	☐ Palaeontology and archaeology
☐	☒ Animals and other organisms
☒	☐ Clinical data
☒	☐ Dual use research of concern
☒	☐ Plants

Methods

n/a	Involved in the study
☒	☐ ChIP-seq
☒	☐ Flow cytometry
☒	☐ MRI-based neuroimaging

Antibodies

Antibodies used

chicken anti-GFP antibody (Invitrogen, A10262, 1:1000; RRID: AB_770014); rabbit V5-Tag (D3H8Q) antibody (Cell Signaling Technology, 13202, 1:2000; RRID: AB_2687461); mouse anti-b-actin antibody (Millipore Sigma, A5441, 1:5000; RRID: AB_476744); anti-mouse (LI-COR, IRDye 680RD, 926-68070; RRID: AB_10956588) and anti-rabbit (LI-COR IRDye 800CW, 926-32211; RRID: AB_621843) fluorescent antibodies; mouse anti-NeuN antibody (Abcam, ab104224, 1:500; RRID: AB_10711040); goat anti-rabbit Alexa Fluor 488 (Invitrogen, A-11034, 1:1000; RRID: AB_2576217); goat anti-mouse Alexa Fluor 568 (Invitrogen, A-11004, 1:1000; RRID: AB_2534072) secondary antibodies; goat-anti chicken Alexa Fluor 488 (Invitrogen, A-11039, 1:1000; RRID: AB_142924) secondary antibodies.

Validation

RRID: AB_770014 (Caldwell et al., Brain Struct Funct, 2023, doi:10.1007/s00429-023-02695-y). RRID: AB_2687461 (Ruiz-Velasco et al., iScience, 2023, 26, https://doi.org/10.1016/j.isci/2023/106970). RRID: AB_476744 (Kim et al., Nat Comm, 2020, 11, 612). RRID: AB_10956588 (Davis et al., iScience, 2023, 26, https://doi.org/10.1016/j.isci.2023.107539). RRID: AB_621843 (Yang et al., J Clin Invest, 2023, 133:e167693, doi: 10.1172/JCI167693). RRID: AB_1071104 (Cho et al., Mol Brain, 2023, 16:2, doi:10.1186/s13041-022-00990-z). RRID: AB_2576217 (Vainorius et al., Nat Comm, 2023, 14:5341, doi:10.1038/s41467-023-40803-y. RRID: AB_2534072 (Fagen et al., Front Aging Neurosci, 2023, 15:1179086, doi: 10.3389/fnagi.2023.1179086). RRID: AB_142924 (Altburger et al., Front Neuroanat, 2023, 17:1196868, doi: 10.3389/fnana.2023.1196868).

Animals and other research organisms

Policy information about studies involving animals; ARRIVE guidelines recommended for reporting animal research, and Sex and Gender in Research

Laboratory animals

Mice were housed at 68-79 deg F and 30-70% relative humidity. C57BL/6, mWake-V5, mWake-Cre, mWake-null, mWake-flox, mWake-Nmf9, Bmal1-, Vglut2-Flp, and Vgat-Flp mice were used in this study. All animals were either generated in C57BL/6 background or backcrossed to C57BL/6 at least seven times. All mice were 2-4 months old and male, except for mWAKE-V5/V5 mice used for DMH neuron counting experiments (which were 2-4 month old female mice), scSEQ experiments (which were 7 week old male mice), patch-clamp recording experiments (which were 5-10 week old male mice), and intersectional validation experiments (which were 2-4 month old female mice).

Wild animals

No wild animals were used in the study.

Reporting on sex

Gender of animals is indicated.

Field-collected samples

No field samples were used in the study.

Ethics oversight

Johns Hopkins IACUC

Note that full information on the approval of the study protocol must also be provided in the manuscript.
